# *Lactobacillus* sp. for the Attenuation of Metabolic Dysfunction-Associated Steatotic Liver Disease in Mice

**DOI:** 10.3390/microorganisms12122488

**Published:** 2024-12-03

**Authors:** Titilayo Olotu, Jessica M. Ferrell

**Affiliations:** 1 Department of Integrative Medical Sciences, Northeast Ohio Medical University, Rootstown, OH 44272, USA; tolotu@neomed.edu; 2 School of Biomedical Sciences, Kent State University, Kent, OH 44242, USA

**Keywords:** MASLD, probiotic, liver disease, steatosis, dysbiosis, *Lactobacillus*

## Abstract

Probiotics are studied for their therapeutic potential in the treatment of several diseases, including metabolic dysfunction-associated steatotic liver disease (MASLD). Part of the significant progress made in understanding the pathogenesis of steatosis has come from identifying the complex interplay between the gut microbiome and liver function. Recently, probiotics have shown beneficial effects for the treatment and prevention of steatosis and MASLD in rodent models and in clinical trials. Numerous studies have demonstrated the promising potential of lactic acid bacteria, especially the genus *Lactobacillus*. *Lactobacillus* is a prominent bile acid hydrolase bacterium that is involved in the biotransformation of bile acids. This genus’ modulation of the gut microbiota also contributes to overall gut health; it controls gut microbial overgrowth, shapes the intestinal bile acid pool, and alleviates inflammation. This narrative review offers a comprehensive summary of the potential of *Lactobacillus* in the gut-liver axis to attenuate steatosis and MASLD. It also highlights the roles of *Lactobacillus* in hepatic lipid metabolism, insulin resistance, inflammation and fibrosis, and bile acid synthesis in attenuating MASLD.

## 1. Introduction

Metabolic dysfunction-associated steatotic liver disease (MASLD, previously referred to as non-alcoholic fatty liver disease/NAFLD) is now recognized as a global public health challenge and is a leading worldwide cause of liver failure [1]. MASLD is a progressive disease that begins with steatosis and can develop into metabolic dysfunction-associated steatohepatitis (MASH), hepatic cirrhosis, or hepatocellular carcinoma [2]. 20% of individuals with MASLD are categorized as having MASH [3]. It is estimated that 18.2 million American adults have Type 2 diabetes (T2DM) and MASLD, and this burden will cost almost $56 billion within the next 20 years, making the global epidemic of MASLD and comorbid disorders a cause for substantial clinical and economic strain in the United States [4]. The pathophysiology of the disease was first described with a “two-hit” hypothesis; the first hit was hepatic steatosis associated with insulin resistance, and the second hit involved inflammatory cascades and fibrogenesis. The “multiple hit” hypothesis is now accepted and better reflects MASLD pathology; multiple insults (including but not limited to obesity with adipocyte proliferation, insulin resistance, oxidative stress, gut dysbiosis, and inflammation) act together to influence genetic and environmental factors that contribute to MASLD [5].

In recent years, there has been landmark progress in MASLD research, and the complexity involved in its pathophysiology is now better understood. The detrimental effects of unhealthy lifestyles are recognized, especially in terms of diet, and several drugs to treat MASLD have been tested in pre-clinical and clinical trials. More recently, next-generation sequencing technology provided evidence indicating the impact of the gut microbiome in the pathogenesis and mitigation of MASLD and its influence on host homeostasis. The gut microbiota consists of trillions of microorganisms (bacteria, fungi, viruses, and other microbes) that inhabit the digestive tract. Modulation of the gut microbiota by dietary factors and the role of the gut microbiota in attenuating MASLD have been reported [6]. *Lactobacillus* is a lactic acid-producing genus and is ubiquitously present within the gut. It has also been extensively used to demonstrate the beneficial effects of probiotics in ameliorating MASLD in mouse models and clinical trials. This narrative review provides a comprehensive summary of the impact of *Lactobacillus* species on bile acid synthesis, lipid and glucose metabolism, inflammation and fibrosis to attenuate MASLD.

### 1.1. Pathogenesis of MASLD

**Figure 1:**
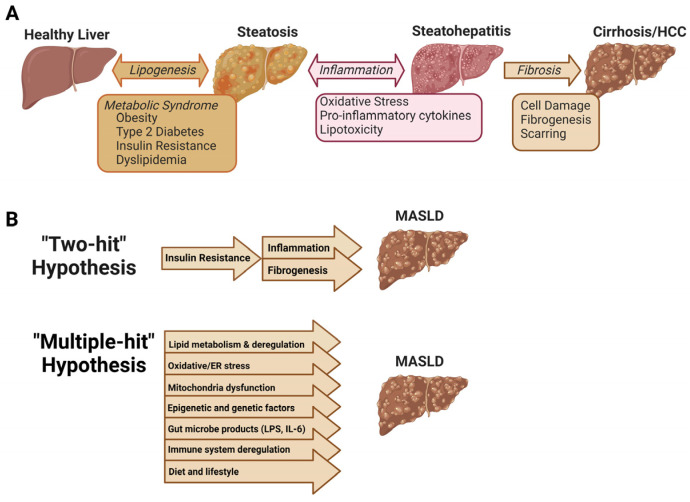
Progression and pathogenesis of MASLD. (**A**). MASLD is a progression of liver disorders beginning with steatosis that can result from obesity or other consequences of metabolic syndrome. Steatosis can progress to inflammatory steatohepatitis due to oxidative stress, inflammatory cytokines, or lipotoxicity in the liver. Both steatosis and hepatitis are considered reversible conditions, though a subset of patients may progress to irreversible cirrhosis or hepatocellular carcinoma (HCC). (**B**). The “two-hit” hypothesis proposed that MASLD progressed first as steatosis due to insulin resistance, followed by inflammatory hits that led to hepatitis and fibrosis. The “multiple-hit” hypothesis recognizes that MASLD progression may involve simultaneous factors, including excessive lipid accumulation, oxidative stress, inflammation, and mitochondrial dysfunction, as well as gut microbe products, epigenetic factors, and lifestyle.

MASLD has a global prevalence of about 30%, with the highest prevalence in South America (31%), Asia (27%), the United States (24%), and Europe (23%), where approximately 20% of individuals with MASLD progress to MASH and fibrosis [7]. Additionally, MASLD progression to MASH, cirrhosis, or hepatocellular carcinoma is predicted to be the most frequent indication for liver transplant by 2030 [8]. The progression of MASLD pathogenesis has been extensively reported, but the mechanisms are not fully characterized (Figure 1) [9]. MASLD development encompasses lipid accumulation, lipotoxicity, endoplasmic reticulum stress, and oxidative stress. Sources of hepatic steatosis include high calorie/high-fat diet, increased adipose tissue proliferation, and hepatic de novo lipogenesis [10,11]. Progression from steatosis to MASH involves complex interactions between parenchymal and nonparenchymal cells in the liver and includes pathological signals from the gut and adipose tissue. Pathological stimuli such as hepatocyte death, adipokines and cytokines, and intestinal pathogens activate Kupffer cells (hepatic resident macrophages) to promote inflammation. Inflammation then leads to further activation of hepatic stellate cells and excessive extracellular matrix synthesis, contributing to scar formation and liver fibrosis [9].

### 1.2. “Multiple Hit” Hypothesis of MASLD

The expansion from the two-hit theory to the multiple-hit theory was proposed upon discovering that hepatic steatosis may be protective against inflammatory effects and the fact that it can lead directly to fibrosis without overt features of the second hit [12]. These conditions are compounded by additional insults from oxidative stress, hepatic inflammatory cytokines, apoptosis, and gut microbe-derived lipopolysaccharide (LPS). Further, steatosis does not always precede inflammation, and MASH may present with or without steatosis. Hepatocyte stress from inflammation may cause hepatic lipid deposition, which implies that inflammation may precede steatosis rather than steatosis necessarily causing inflammation; this is proposed as the distinct-hit theory [5,12].

## 2. Metabolic Pathways Altered by Dysbiosis in MASLD

Dysbiosis is an imbalance in gut microbiota composition, a common feature in MASLD. The gut microbiota comprises bacteria, archaea, fungi, and viruses, with bacteria being the most abundant component. These organisms play roles in energy harvesting, nutrient digestion and metabolism, immune modulation, protection against pathogens, and microbial toxin production. Specific microbial populations ferment dietary fiber to produce short-chain fatty acids (SCFA), contribute to vitamin synthesis (vitamins B and K), metabolize sterols and bile acids, and produce hormones that influence host metabolism [13,14]. Gut microbiota dysbiosis can contribute to the pathogenesis and severity of MASLD via the reduction and/or loss of a specific group of microbial species or overgrowth of pathogenic species [15].

Unhealthy lifestyles, especially chronic consumption of high-fat or high-sugar diets, promote dysbiosis that can lead to the development of MASLD via altered gut-liver axis homeostasis. Gut microbiome dysbiosis significantly disrupts intestinal barrier function, decreases SCFA, and alters bile acid synthesis and composition; it can also increase bacterial endotoxin production [16]. One such endotoxin, LPS, was correlated with inflammatory MASLD in human patients and in mice compared to those with simple steatosis [17]. Upregulation of serum and hepatic LPS, p-NF-κB and Toll-like receptor 4 (TLR4+) macrophages in patients with MASH were also increased compared to patients with simple steatosis and healthy controls. Likewise, MASH model mice had increased serum and hepatic LPS associated with upregulation of NF-κB activation [17].

### 2.1. Intestinal Barrier Function

**Figure 2:**
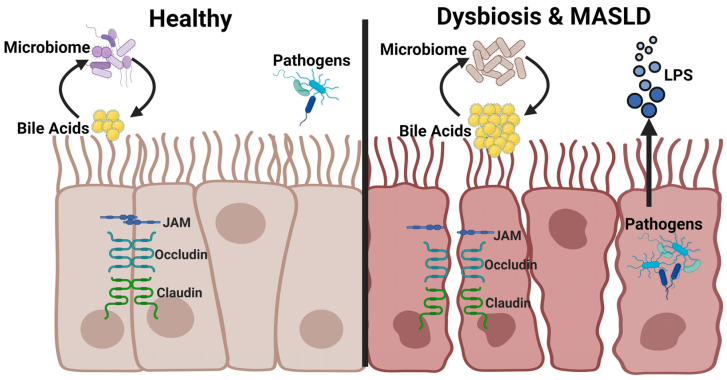
Physiological changes to the intestinal barrier in MASLD. (**Left**) In a healthy gut, tight junction proteins (JAM, Occludin, and Claudin) maintain the intestinal barrier and prevent pathogenic infiltration into enterocytes, while bile acid content is maintained through a bi-directional relationship with the gut microbiota. (**Right**) In MASLD, tight junctions are disrupted and may allow pathogenic invasion that releases systemic LPS. Bile acid content may be increased, and composition may be altered, in part due to dysbiosis resulting from loss of biodiversity of the microbiota.

The intestinal barrier is a physical and functional barrier that prevents the translocation of harmful luminal antigens into the systemic circulation; it consists of mechanical, chemical, microbial, and immunological barriers (Figure 2). The mechanical barrier is composed of the epithelial layer, mucus layer, tight junctions (TJ), and adherens junctions (AJ). This layer is the first line of defense against pathogens, microbes, and their products, whereby the gut mucosa prevents harmful contents from entering the systemic circulation. TJs are composed of transmembrane proteins like claudins and intracellular scaffold proteins like zonula occludens (ZO-1, -2, and -3), and they link TJs to the actin cytoskeleton [18,19]. AJs perform several functions, including cell-cell stabilization, adhesion and transcriptional regulation. AJs are transmembrane glycoproteins that include E-cadherin, α-catenin, β-catenin, and p120-catenin [20].

The chemical barrier comprises digestive enzymes, antimicrobial peptides, mucus, bile acids and other molecules. Gastric acid prevents microbial colonization by separating bacteria from the intestinal tract [21]. Mucins are the primary building blocks of the mucus gel of the intestinal mucosa; they form a protective physical barrier preventing microorganisms and harmful substances from translocating to the surface of the epithelium [22]. Goblet cells located in the intestinal mucosa secrete Muc2, Muc5AC, and Muc6 proteins [23]. *Muc2*^−/−^ mice have significantly increased colonization of enteric pathogens compared to control wild-type mice [24]. Cytokines are activated by their contact with pathogen-associated molecular patterns (PAMPs), and several regulate mucin synthesis, including interleukin *(IL)-1β*, *1L-13*, *IL-4*, *1L-13*, and tumor necrosis factor α (*TNFα*) [22].

Muc2 deficiency protected mice from high-fat diet (HFD)-induced fatty liver disease and obesity. These mice also exhibited improved glucose homeostasis, reduced inflammation, and upregulated expression of genes involved in lipolysis and fatty acid β-oxidation in white adipose tissue. The absence of Muc2 likely decreased the content of the mucus layer; this breach in the mechanical barrier allowed better access to immune cells, which primarily initiated intestinal wound healing and restored barrier dysfunction [23]. The translocation of pathogens through the intestinal mucosa barrier is recognized and eliminated by immune cells acting through receptors, including TLRs and nucleotide-binding oligomerization domain receptors. These receptors recognize microbe-produced PAMPs, thus triggering an immune response to release cytokines [25]. The liver is a unique immune environment exposed to various immunogenic factors from the gastrointestinal tract, including components of pathogenic bacteria and viruses; the presence of these factors leads to inflammatory consequences such as cell damage, organ dysfunction, fibrosis, and carcinogenesis [26]. With obesity and steatosis, inflammation can occur in the liver, pancreas, and muscle tissue and is associated with insulin resistance and metabolic dysfunction. Disequilibrium between pro- and anti-inflammatory cytokines affects β-oxidation, lipid transport and storage, insulin and nuclear receptor signaling, autophagy, and eventually progression of MASLD to MASH [27].

Antimicrobial peptides protect against pathogens to prevent disruption and dysfunction of the intestinal barrier. Bile acids control bacteria growth and protect against pathogens. They also play a pivotal role in glucose, lipid, and energy homeostasis. Meanwhile, immune cells prevent microbes, pathogens, and antigens from moving into the luminal space [28]. Several studies have linked dysbiosis with increased intestinal permeability associated with decreased Firmicutes: Bacteroidetes ratio and increased Escherichia, Proteobacteria, and Enterobacteria that can increase dangerous microbial metabolites that mediate MASLD progression [29]. The intestinal mucosa and associated epithelial cells are junctional complexes of the intestinal mechanical barrier. The destruction of these junctional proteins causes intestinal inflammation and alters the integrity of the intestinal barrier, factors important in developing MASLD [30]. Decreased ZO-1 and occludin expression was observed in MASLD patients with increased levels of transaminase [29,31]. HFD-fed mice with a loss of junctional proteins showed increased intestinal permeability, affecting the gene that encodes the TJ protein Jam-a [32]. A decrease in Jam-a expression in the mouse colon caused an increase in inflammatory proteins that led to steatohepatitis progression [30]. A meta-analysis report indicated MASLD patients had increased intestinal permeability and altered gut microbiota that promoted microbiome translocation compared to healthy controls [33].

Increased gut permeability is often associated with MASH patients, who demonstrate a decrease in JAM-A and ZO-1 proteins [32,34]. In a mouse model of colitis, a leaky and inflamed gut barrier increased systemic LPS, which worsened hepatic inflammation and fibrosis in HFD-fed mice [35]. Patients with MAFLD have reduced T regulatory cells and increased Th1 and CD8+ T cells in the lamina propria [36]. Molecules from immune cells like histamine, proteases, and cytokines impact intestinal barrier function. Proteases, tryptase, and chymase can lead to the cleavage of ZO-1, downregulation of JAM-A, and increase the epithelial permeability and gut bacterial translocation [37]. Thus, the intestinal barrier plays a significant role in nutrient absorption and homeostasis, preventing pathogenic metabolites and endotoxin absorption. Conversely, a compromised intestinal barrier can cause systemic inflammation, increase lipid accumulation, and accelerate obesity and fatty liver diseases [37].

### 2.2. Bacterial Endotoxins

The liver is the first systemic barrier between gut pathogens and their products due to first-pass portal circulation. Intestinal barrier dysfunction increases intestinal permeability and allows PAMPs to translocate to the liver, leading to hepatic inflammation. PAMPs include LPS, peptidoglycan, viral nucleic acids, or fungal cell wall components, and they function to activate the hepatic immune response and induce inflammation [38]. LPS is a main component of Gram-negative bacteria cell walls, is recognized by the immune system via TLR4, and induces hepatic inflammation [39]. HFD consumption leads to excessive uptake of free fatty acids (FFA), which causes gut dysbiosis and increases bacterial products. LPS translocation through the gut barrier activates hepatic stellate cells (HSCs) and Kupffer cells in the liver and activates MyD88, which induces transcription of NF-κB and produces TNF-α, IL-1, IL-6, and other cytokines [40].

Inflammation is a major contributor to the progression of MASLD to MASH and beyond. Many studies have reported elevated LPS concentrations in obese individuals compared to lean individuals due to an altered gut microbiome [39]. Refs. [41,42] elevated serum LPS was observed, which positively correlated with steatosis and inflammation in patients with MASLD compared to healthy individuals. LPS is a contributor to the progression of MASLD because of its ability to activate hepatocyte inflammation. However, it was found that obese individuals had increased circulating lipopolysaccharide-binding protein (LBP) but had a more protective metabolic profile and lower MASLD prevalence. It was proposed that this may have resulted from chronic lipid deposition that enhanced the degradation of hepatic LPS, protecting their metabolic profile [43]. LPS is recognized by TLR4, LBP and cluster of differentiation 14 (CD14). Decreased lipid accumulation, hepatic inflammation, and injury are seen in Tlr4-deficient mice compared with wild-type mice given a high fructose diet, affirming the role of TLR4 in MASLD. CD14 mutant mice are resistant to metabolic dysfunction induced by LPS, indicating that they may trigger MASLD in a CD14-dependent manner [44]. Ref. [45] observed that increased serum IL-18 correlates with greater hepatic injury and that it can activate chemotactic responses, causing infiltration of inflammatory cells. IL-18 may activate JNK-1 signaling in adipose tissues to cause hepatic steatosis and inhibit insulin signaling [46]. Increased TNF-α can activate Kupffer cells, releasing proinflammatory cytokines and causing hepatocyte apoptosis, and TNF-α can aggravate mitochondria dysfunction that increases ROS production and lipid peroxidation [45]. TNF-α is produced by adipocytes, stimulates hormone-sensitive lipase and insulin-dependent glucose metabolism, and is associated with increased FFA in obese patients with MASLD. Kupffer cells in the liver generate cytokines that produce TNF-α in response to bacterial toxins, a process mediated by TLRs [47].

### 2.3. Short Chain Fatty Acids

Gut dysbiosis or abundance of pathogenic species increases microbe-associated molecular patterns (MAMPs) and LPS into the systemic circulation, activating TLRs in liver cells to promote inflammation and fibrosis [48]. The gut microbiota is also composed of beneficial genera such as *Lactobacillus*, *Bifidobacterium*, *Akkermansia*, and many others that generate several key metabolites like SCFAs, bile acids, phenolics, and carotenoids needed to regulate intestinal metabolism and prevent inflammation [49]. SCFAs are produced from fermented indigestible dietary fibers and are the most abundant microbial metabolites produced by intestinal bacteria. These include butyrate, propionate, and acetate, and they play critical roles in immune responses, glucose homeostasis, lipid metabolism, and appetite regulation [50]. Butyrate enhances insulin signaling, exerts anti-inflammatory effects, is involved in energy metabolism, and improves leptin gene expression, and is thus considered a beneficial metabolite [51]. Propionate regulates appetite by controlling anorexigenic peptides like glucagon-like peptide-1 (GLP-1) and peptide YY; it also reduces the activity of enzymes involved in de novo fatty acid and cholesterol synthesis and is involved in hepatic gluconeogenesis [50]. Acetate in systemic circulation influences muscle, adipose tissue, and the brain; it stimulates lipids synthesis in the liver, prevents dyslipidemia, and induces insulin and ghrelin secretion release [52]. Both animal and human data have shown that acetate plays a crucial role in reducing pro-inflammatory cytokines. This action, in addition to its stimulation of gut hormone secretion, influences host energy and substrate metabolism. Furthermore, the impact of acetate on metabolism extends to appetite regulation, and it promotes whole-body lipolysis by increasing fat oxidation and energy expenditure [53]. Acetate can act by binding to G-protein coupled receptors GPR43 (FFAR2) and GPR41 (FFAR3) in the human colon [54,55] and in the small intestine, particularly the ileum [56]. Acetate may be converted to acetyl-CoA and incorporated in the tricarboxylic acid (TCA) cycle in various peripheral tissues [53], and it can impact oxidative capacity in liver and skeletal muscle through adenosine monophosphate-activated protein kinase (AMPK) phosphorylation [57].

SCFAs promote lipid oxidation and attenuate insulin resistance via an AMPK-dependent mechanism. SCFAs maintain TJ integrity, decrease LPS concentration in systemic circulation, negatively regulate NF-κB and decrease secretion of pro-inflammatory factors [58]. SCFAs are altered with MASLD, but how these changes relate to disease progression is still unclear. Some studies report a reduction of plasma SCFAs in hepatic fibrosis or cirrhosis, and SCFA-producing bacterial phyla are significantly reduced in advanced stages of cirrhosis [59,60]. It was also reported that fecal SCFAs were increased in patients with MASLD compared with healthy individuals. Ref. [61] found an increase in plasma butyrate, acetate, and propionate that was not significant in MASLD compared with healthy individuals but a significant decrease in MASH compared with MASLD patients and lower butyrate concentration in MASLD-cirrhosis compared with MASH patients. The role of SCFAs in MASLD is a topic that still requires further investigation. The contradictory results of clinical studies underscore the need for more comprehensive and evaluative observations [50]. This also highlights the importance of ongoing research in this area and the potential for new insights to be gained. Studies may involve diverse populations with varying genetic backgrounds, dietary habits, and gut microbiota compositions, which might lead to some contradictory observations. Some studies found no clear differences between controls and patients with MASLD, especially in plasma SCFAs, which were elevated in MASLD patients but not significantly [61]. Refs. [61,62,63] observed statistically reduced plasma SCFAs in MASH and cirrhosis individuals, which was attributed to elevated TNF-α. Obese individuals have altered fecal SCFAs, particularly propionate, and fecal SCFA concentrations are inversely correlated with microbial diversity and obesity [64]. Likewise, overweight and obese individuals have higher fecal SCFA concentrations than lean individuals [65], and the excessive production of SCFAs may contribute to weight gain due to increased energy storage [66].

Study discrepancies may reflect differences in study design, such as the procedures used for selecting control and MASLD patients and the severity of the underlying MASLD. However, potential mechanisms underlying its activity still need to be assessed in the context of its gut microbiota modulation [67]. Reported clinical trials summarize that patients with MASLD exhibit a significantly reduced abundance of SCFA-producing bacteria such as *Bacteroides*, *Lactobacillus curvatus*, and *Lactobacillus plantarum* [66,68]. Individuals with obesity and MASLD tend to have higher levels of fecal SCFAs because of reduced absorption of SCFAs in the colon, leading to higher fecal concentrations of SCFAs [66]. SCFAs exhibit therapeutic potential in preventing and managing MASLD by targeting pathways involved in its pathogenesis. Supplementation with SCFAs (sodium acetate and sodium butyrate) in mice models protects against hepatic steatosis [69]. Butyrate supplementation increased GLP-1 receptor expression in patients with MASLD, and mouse studies indicate this may occur via altered histone acetylation that promotes energy metabolism [70]. Butyrate activates AMPK to induce the expression of fatty acid oxidation genes in hepatocytes, improving insulin sensitivity and thereby reducing steatosis [71,72]. Together, these studies indicate that probiotics may act via several beneficial pathways involving SCFAs to improve MASLD.

### 2.4. Altered Bile Acid Synthesis and Composition

Bile acids (BAs) are synthesized from cholesterol in the liver. This is initiated by the rate-limiting enzyme cholesterol 7α-hydroxylase (CYP7A1), which converts cholesterol to the primary bile acids cholic acid (CA) and chenodeoxycholic acid (CDCA). BAs facilitate lipid digestion and absorption; they also act as signaling molecules via receptors TGR5 (Takeda G protein-coupled receptor 5) and farnesoid X receptor (FXR) that regulate glucose, lipid, and energy metabolism as well as sensors that regulate BA synthesis and transport. The circulating BA composition determines the activation of these receptors as well as efficiency in lipid absorption [44]. Importantly, the primary bile acids synthesized in the liver are biotransformed to secondary bile acids by gut microbes. Aside from the regulation of glucose, lipids, and energy metabolism, bile acids exhibit anti-microbial properties in the gastrointestinal environment [73].

Therefore, bile acids and the gut microbiota exist in a complex, bi-directional regulatory relationship. Liver and serum bile acids may be elevated with MASLD, though the evidence is conflicting [44]. Bile acid hydrophobicity, determined by the number and position of hydroxyl groups on cholesterol, may also be increased with MASLD [45]. Hydrophobic bile acids are more toxic and stronger detergents but also exhibit greater antimicrobial capacity. In this way, bile acids can shape gut microbiota composition and maintain gut homeostasis. Altered bile acid pool size and composition can aggravate inflammation and the abundance or activity of opportunistic pathogens [74]. Altered bile acid homeostasis affects hepatic metabolic homeostasis and may reshape the gut microbiome, causing dysbiosis, inflammation and enhanced pathogenesis of metabolic disorders, including MASLD, obesity, T2DM, and inflammatory bowel disease [75].

Therefore, ameliorating dysbiosis is a promising treatment strategy [74]. Dysbiosis alters microbial populations that transform bile acids, increasing primary bile acids and decreasing secondary bile acids, and this decrease is associated with a reduction in the abundance of bile salt hydrolase (BSH)-producing bacteria [76]. BSH bacteria that inhabit the small intestine and colon can convert primary bile acids (CA and CDCA) to the secondary bile acids lithocholic acid (LCA) and deoxycholic acid (DCA), and their activities shape the BA pool [77]. Altered BA synthesis, composition, and metabolism are associated with MASLD and other metabolic diseases, and numerous studies have reported elevated serum and hepatic BA levels in patients with MASLD. In MASLD, BA composition changes substantially with increased TBA (total bile acids), though some studies observed no changes in TBA. Increased TBA, primary BAs, and secondary BAs were observed in patients with MASH [78,79]. Patients with MASH have elevated levels of TBA, both in liver tissue and plasma, suggesting a relationship between toxic levels of BA and the development of MASH [80]. Altered plasma BA levels have been reported in obesity and obesity-related diseases. MASLD/MASH might cause a shift in BA synthesis; mRNA levels of CYP7B1 are increased, and CYP8B1 expression levels were decreased in patients with MASLD [81,82]. As MASLD progresses and tissue damage increases, the BA synthesis and secretion abnormally increase. Simultaneously, a reduced flow that often occurs with hepatocyte failure will lead to accumulation. Subsequently, this altered BA composition can further liver injury [82]. Dysregulation of bile acid metabolism may cause liver-related diseases, such as cholestasis, T2DM, MASLD, MASH and HCC etc. [83]. Bile acid content may influence MASLD progression, and elevated serum bile acids that correlated to steatosis severity were associated with MASLD [84]. Bile acid composition and pool size are altered in insulin-resistant patients, characterized by increased hydrophobic plasma bile acids and 12α-hydroxylated bile acids. The postprandial increase in serum insulin and glucose levels is correlated with increased bile acid synthesis and serum GLP-1 and triglyceride levels [85]. Finally, several human and animal studies report increased TBA levels associated with MASLD and obesity because of alterations in the circulating BA profiles [86,87].

### 2.5. Increased Energy Harvest

Dysbiosis alters the gut Bacteroidetes/ Firmicutes ratio, shifting in favor of Firmicute, which is associated with increased energy harvest, which leads to weight gain, obesity, and T2DM [88]. Species belonging to Firmicutes extract energy from food more effectively than Bacteroidetes and allow the host to absorb more calories [89]. Fat deposition in patients with steatosis is linked with an altered gut microbiome that results in enhanced energy production compared to lean patients. Metagenomic and proteomic analysis of the cecal microbiome of obese children identified more pathways devoted to energy production and harvesting that likely provide more energy to the host. This reason may explain why obese children gain more weight than healthy children placed on the same diet [90]. This study is similar to the observation of [91], where the microbiota of obese mice was more enriched with enzymes involved in carbohydrate digestion compared to lean mice. Ref. [89] compared the metabolic pathways of the microbiome of lean and obese twins and found that those with obesity had enriched the microbial processing of carbohydrates. Germ-free mice transplanted with the gut microbiome from individuals with obesity gained more weight compared to lean mice with the same food intake. An obesity-associated microbiome is associated with an increased Firmicutes: Bacteroidetes ratio and reduced microbial diversity [89]. Obese children may have reduced *Bacteroides* species and reduced Bacteroidetes: Firmicutes ratio that negatively correlates to BMI [92].

### 2.6. MASLD and Sarcopenia

Progressive MASLD also exerts extra-hepatic effects, including sarcopenia or muscle loss, resulting from hepatocarcinoma [93]. A recent systemic review reported that nearly 40% of patients with HCC also had sarcopenia, which was independently associated with reduced overall survival [94]. Interestingly, sarcopenia is also associated with gut dysbiosis and the administration of probiotics, including *Lactobacillus* and other genera, significantly improved muscle mass and muscle function as analyzed in a meta-analysis of 17 studies [95]. The mechanisms are not known but may involve increased activation of AKT and modulation of NF-κB and gut-produced cytokines [96,97].

## 3. *Lactobacillus* for the Treatment of MASLD

Probiotics are often referred to as “good bacteria” that can benefit the gastrointestinal tract by reducing the symptoms discussed above or by outcompeting harmful species [98]. Microbiota studies in humans have revealed that gut dysbiosis is associated with diverse liver and inflammatory diseases, metabolic disorders, and colorectal cancer [99]. Probiotics predominantly belong to the phyla Firmicutes, Bacteroidetes, Proteobacteria and Actinobacteria [100] and can benefit the host’s physiological functions while the host provides a favorable habitat and nutrients. Some probiotic bacteria defend against pathogens by secreting antimicrobial peptides that inhibit or compete for nutrition and adhesion sites with pathogens, thereby destroying them [101]. One of the significant benefits of probiotics is preventing the production of LPS or its uptake in the gut and reducing inflammatory activity. Immune stimulation of dendritic cells prevents pathogen translocation and strengthens immunological status. These activities influence hepatic fat metabolism and enhance liver function [102]. With the increasing scientific evidence that probiotics benefit human health, *Lactobacillus* has emerged as a promising treatment to ameliorate MASLD and other gut disorders [103].

### 3.1. Mechanisms of Action of Lactobacillus spp.

**Figure 3:**
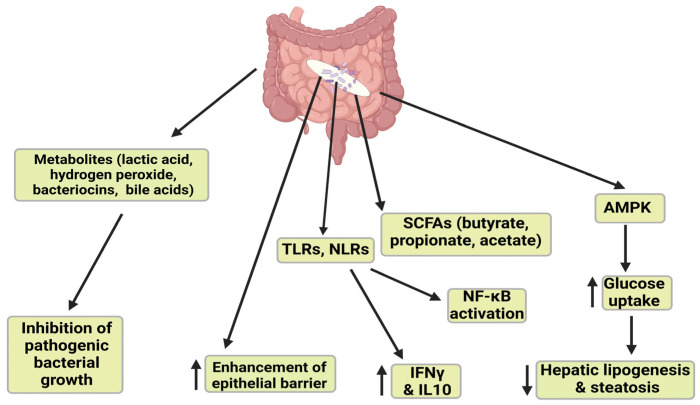
Mechanism of action of *Lactobacillus* spp. *Lactobacillus* spp. ferments dietary fibers to produce SCFAs, SCFAs can activate AMPK, enhance fatty acid oxidation and improve insulin sensitivity. Steatosis is reduced through the inhibition of key lipogenic enzymes. *Lactobacillus* spp. competitively excludes opportunistic pathogens and restrains their attachment to the epithelium by producing metabolites like lactic acid, hydrogen peroxide, and bacteriocins and competing with binding sites with these pathogens. *Lactobacillus* strains strengthen the gut barrier by enhancing mucus production that prevents the translocation of harmful bacteria and toxins into the bloodstream. These strains also bind to epithelial cells to activate immune cells, including dendritic cells and macrophages, to produce anti-inflammatory cytokines like IFNγ and IL10. Finally, *Lactobacillus* MAMPs bind to TLRs, leading to modulation of NF-κB, which is involved in immune and inflammatory responses.

*Lactobacilli* are lactic acid-producing bacteria (LAB); LAB are found in fermented foods, and they are considered safe bacteria with a long history of beneficial effects [104]. *Lactobacilli* are Gram-positive, facultative rod-shaped anaerobic bacteria that prefer microaerophilic conditions. *Lactobacilli* have a high tolerance to low pH and high bile salt concentrations, can survive intestinal enzymes, and have antioxidant and antimicrobial properties. The main strains of *Lactobacillus* species are *L. acidophilus*, *L. johnsonii*, *L. casei*, *L. helveticus*, *L. rhamnosus*, *L. paracasei, L. brevis*, *L. plantarum*, *L. fermentum*, *L. gasseri*, *L. delbrueckii* subsp. *Bulgaricus* and *L. reuteri*. LABs are known to stimulate the immune system, aid in the digestion of lactose, ameliorate diarrhea, modulate the gut microbiome, and have antimicrobial and anti-cancer properties [104]. *Lactobacillus* can impact gut health via several pathways, including modulating the growth of other microbiota species, strengthening the intestinal barrier by increasing mucus production, modulating the immune system and maintaining immune homeostasis, and reducing inflammation by mediating the release and degradation of inflammatory factors. *Lactobacillus* can communicate with the brain and other organs through the microbiota-gut-brain/liver/lung axes [105]. Specifically, *Lactobacillus* competes with harmful bacteria for nutrients and adhesion sites on the gut lining, preventing the establishment and proliferation of pathogenic bacteria; they produce metabolites like lactic acid, hydrogen peroxide, and bacteriocins that inhibit pathogenic bacteria growth. *Lactobacillus* strains strengthen the gut barrier by enhancing mucus production, which prevents the translocation of harmful bacteria and toxins into the bloodstream. *Lactobacillus* also modulates the immune system by enhancing immune cell activity, promoting the production of anti-inflammatory cytokines, and suppressing pro-inflammation cytokine release [106]. SCFAs inhibit the activation of NFκB and promote the production of anti-inflammatory cytokines [66]. Numerous studies have reported the ability of LAB to survive acidic conditions, high bile salt concentration environments, and gastrointestinal conditions while maintaining colonization near epithelial cells [107,108]. Lactate and other metabolites like short-chain fatty acid (SCFA) produced by *L. acidophilus* are activators of AMPK, enhancing glucose uptake and fatty acid oxidation [109]. AMPK induces Glut 4 to enhance glucose uptake, and this inhibits the synthesis of fatty acids and cholesterol that downregulates insulin. Lactic acid bacteria are known for immune modulation by inhibiting the TLR4/NF-KB pathway and stimulating anti-inflammatory cytokines [110]. *L. acidophilus* is expected to survive the gastric, duodenal, and intestinal fluid, and it is expected to exhibit strong scavenging potentials through its ability to neutralize free radicals and reactive oxygen species [111]. *Lactobacilli* are potential adjuvants triggering mucosal and systemic immune responses [105] via increased natural killer cell cytotoxicity to target and eliminate harmful cells, inducing interferon-γ production. As a result, immune responses and cytokine expression are enhanced to regulate the immune system [105]. *Lactobacilli* adhere to the host’s intestinal epithelium to exert immunomodulatory effects. *Lactobacilli*, especially *L. acidophilus*, administered with bifidobacteria, enhance the immune system through systemic/local immunity and concurrently attenuate systemic stress response in a double-blind, placebo-controlled, randomized trial [112]. *Lactobacillus* spp. are bile acid hydrolase LAB that converts primary BA to DCA and LCA, which in turn controls gut microbial overgrowth and shapes the intestinal bile acid pool [113]. These bacteria deconjugate bile salts into free choline, glycine and amino groups by synthesizing bile salt hydrolase. Free choline excreted is absorbed in the intestine, and free taurine and glycine return to the liver. This increases the elimination of bile from the body, and more cholesterol is used to synthesize bile, thereby reducing cholesterol levels in the blood [106]. Several *Lactobacillus* strains have served as therapeutic adjuvants against MASLD and metabolic disorders because they increase microbial richness and diversity, increase enzyme (lactase) production, improve the immune microenvironment, and improve intestinal permeability (Figure 3) [106,114]. Numerous studies have reported the effect of *Bifidobacterium*, *Pediococcus*, and *Lactobacillus* in reducing energy intake, inducing weight loss, alleviating insulin resistance, and improving glucose and lipid metabolism. LAB can impact obesity and related conditions by producing SCFA in the intestine; SCFA may then inhibit lipid synthesis. *Lactobacillus* is the most studied, used, and reported LAB [115]. Evidence indicates that *Lactobacillus* probiotic activity is both species- and strain-dependent [103]. There are fewer clinical studies on probiotics treatment compared to animal studies, and most human clinical trial findings are based on a few parameters of lipid profiling, while microbiota modulation of inflammation is less understood. Based on the beneficial probiotic qualities of *Lactobacillus*, its therapeutic impact on bile acid synthesis, lipid and glucose metabolism, inflammation and fibrosis, intestinal barrier function, SCFAs, and gut microbiota modulation will be summarized below.

### 3.2. Intestinal Barrier Improvement

The diverse impact of probiotics includes antibacterial activity against pathogens, immune modulation, enhanced gut barrier integrity, and modulating energy metabolism, all critical factors in ameliorating MASLD [116]. *Lactobacillus rhamnosus GG* (LGG) protected mice from MASLD induced by a high-fructose diet via reduced duodenal IκB, attenuated LPS, *Il-1β*, and *Tnf*-α, and reduced liver transaminase [117]. The effect of single-strain and combination administration of *Lactobacillus* and *Pediococcus* was tested in mice fed a Western-style HFD. Single-strain groups (*L. helveticus*, *L. casei*, *L. bulgaricus* and *P. pentosaceus*) showed better improvement in liver/body weight ratio, liver function tests and cholesterol levels than combined strains; however, a non-significant improvement in steatosis and inflammation was observed with administration of combined strains. A recent report indicated that the combination of *L. casei*, *L. delbrueckii*, *L. acidophilus*, *Streptococcus salivarius*, *B. breve* and *B. longum* in mice was associated with reduced liver enzymes and body weight. These data support the notion that different species of *Lactobacillus* differentially affect weight, and effects may be host-specific [118,119].

*L. bulgaricus* and *L. helveticus* improved body weight, liver weight, liver function and MASLD, while *L. casei* exhibited greater restoration of the Firmicutes: Bacteroidetes ratio, which is necessary to maintain intestinal homeostasis. HFD reduced TJ protein expression in mouse ileum (Zo-1, claudin 2, and claudin 5), and *L. sakei* WIKIM31 restored levels to that in chow-fed mice. It also reduced intestinal *Il-6*, *Tnf-α*, and monocyte chemoattractant protein-1 mRNA and increased propionate and butyrate [120]. LGG did not change IL-10 expression in HFD-fed mice, but the pro-inflammatory cytokines IL-6 and IL-12 were significantly decreased in serum. Mice without LGG treatment also had increased plasma LPS. LGG protects against inflammation and suppresses the hepatic inflammatory response by regulating the barrier function protein expression [121]. Occludin and claudin-1 expression were significantly reduced in mice fed a high-fructose diet compared to a control diet, and oral treatment with LGG restored the expression of these proteins. In contrast, Zo-1 and 2 protein expression was neither influenced by a high-fructose diet, nor by LGG treatment [117] *L. plantarum* ZLP001 fortifies the intestinal barrier by strengthening epithelial defense functions by maintaining TJ protein abundance [122]. *Lactobacillus* species are believed to enhance intestinal barrier defense by promoting mucus secretion. In vitro studies of *L. casei* T21 in colonic epithelial cells (Caco2 and HT29) challenged with *C. difficile* have demonstrated upregulation of the protective *MUC2* gene [123]. SCFAs increase transepithelial electrical resistance and stimulate the formation of tight junctions in Caco2 intestinal epithelial cells in vitro via inhibition of the NLRP3 inflammasome and autophagy [124]. *L. plantarum* WCSF1 administration into the duodenum of healthy human subjects increased ZO-1 and occludin staining in the vicinity of TJ structures via activation of TLR-2 [103,125]. *L. plantarum* WCSF1 administration into the duodenum of healthy human subjects increased ZO-1 and occludin staining in the vicinity of TJ structures via activation of TLR-2 [103,125,126]. The probiotic VSL#3 also increased expression of ZO-1 protein in a double-blinded study in patients with MASLD compared with the placebo group [127]. In another clinical study of small intestine barrier function, biopsy samples demonstrated that *L. plantarum* strain TIFN101 and, to a lesser extent, *L. plantarum* WCFS1 and CIP104448 modulated an increase in gene expression of TJ and adherens junction proteins in a randomized, double-blind placebo-controlled trial.

### 3.3. Gut Microbiome Modulation

The overall community signature of a gut microbiome is assessed through α- and β-diversity; α-diversity estimates species richness and evenness, while β-diversity compares the diversity between different communities that give insight into how these communities differ in terms of species composition. Clinical trials point to key changes in the microbial composition of MASLD compared to healthy individuals. Firmicutes, Proteobacteria, and Actinobacteria were increased, and *Actinomycetaceae*, *Lachnospiraceae*, *Bacteroidaceae* and *Bacteroidales* were decreased in patients with MASLD and MASH compared with healthy controls [128,129,130]. Another study identified an increased abundance of Proteobacteria, *Escherichia*, *Prevotella*, and *Streptococcus* and decreased levels of *Coprococcus*, *Faecalibacterium*, and *Ruminococcus* in patients with MASLD compared with healthy controls [68,129]. Increased levels of Proteobacteria and *Escherichia* are associated with MASH [131], while increased *Enterobacteriaceae* and *Prevotella* are associated with high body fat percentage and elevated serum levels of LPS and IL-6 compared to healthy controls [132]. Jee et al., 2022 [132] observed *Enterobacteriaceae* and *Prevotella* in serum and fecal samples MASLD patients, increased intestinal viral diversity and bacteriophages, increased *Escherichia*, *Enterobacteria* and decreased *Lactococcus* and *Leuconostoc* phages in fecal and serum samples of MASLD individuals compared with healthy controls. Ref. [133] reported increased Enterobacteriaceae and Proteobacteria, decreased ratio of Firmicutes and Bacteroidetes and a decrease in species *Akkermansia muciniphila*, *Alistipes putredinis*, *Bacteroides uniformis*, *Bacteroides fragilis*, *Oscillibacter* sp. *ER4*, *Ruminococcus bromii* and *Eubacterium ventriosum. Blautia abundance* is associated with MASH but not MASLD or obesity, while increased *Bacteroidaceae* and *Bacteroides* are found with obesity [134]. The gut microbiota in patients with MASLD is characterized by a high abundance of pathogens such as *Escherichia coli*, *Campylobacter jejuni*, *Salmonella enterica*, *Vibrio cholerae*, and *Bacteroides fragilis* [133]. Increased *Enterobacteriaceae* was associated with high body fat percentage, serum levels of LPS and imbalanced gut microbiome based on the abundance of *Prevotella* species in in patients with MASLD [135]. Overall, future research should focus on the identification of unique microbiome signatures of MASLD/MASH, which may include increased *Escherichia*, *Enterobacteria*, and *Prevotella*, and decreased *Lactococcus*, which may serve as both biomarkers for diagnosis and targets for treatment.

Treatment with VSL#3, a probiotic supplement consisting of Bifidobacterium breve, *Bifidobacterium longum*, *Bifidobacterium infantis*, *Lactobacillus acidophilus*, *Lactobacillus plantarum*, *Lactobacillus paracasei*, *Lactobacillus bulgaricus*, and *Streptococcus thermophiles* decreased serum ALT levels and increased the Bacteroidetes–Firmicutes abundance ratio in patients with MASH [136]. Administration of *Lactobacillus sakei* MJM60958 increased the relative abundance of Verrucomicrobia and reduced Firmicutes, which are abundant in MASLD mice [137]. Supplementation with *Lactiplantibacillus plantarum* DSM20174 restored Acetatifactor to levels even higher than usual [138]. Patients with MASH were given a probiotic/prebiotic formulation (consisting of *L. plantarum*, *Lactobacillus delbrueckii* ssp. *bulgaricus*, *L. acidophilus*, *L. rhamnosus*, and *Bifidobacterium bifidum* and prebiotic fructose-oligosaccharides) had decreased serum ALT levels and increased Bacteroidetes–Firmicutes abundance ratio [136,139]. Treatment with *Lactobacillus* spp. restores the Firmicutes/Bacteroidetes ratio and increases *Acetatifactor* (a GLP-1 stimulator). Comparing gut microbiota composition before and after *Lactobacillus* supplementation, tracking *Lactobacillus* strain-to-strain effects in MASLD, and investigating microbial metabolites that result from *Lactobacillus* treatment will assist in developing more targeted probiotic treatments for MASLD. Finally, long-term monitoring of continuous probiotic supplementation will provide an understanding of the permanence of the effects of probiotics on gut health and liver disease.

### 3.4. Bile Acids

*Lactobacilli* are BSH bacteria involved in BA transformation, and BA synthesis represents a major route of cholesterol catabolism [140]. BSH activity is essential to maintaining the BA pool, and its function is critical in shaping the intestinal bacteria niche that inhabits the GI tract. Enhanced BSH function is associated with *Lactobacilli* [140]. Hypercholesterolemia involves high plasma levels of both total cholesterol and low-density lipoprotein (LDL), with decreased high-density lipoprotein (HDL) [141]. BSH-active bacteria is characterized as a leading candidate for preventing hypercholesterolemia [25,142]. Studies have reported probiotics with high BSH activity, including *Lactobacillus* (*L. reuteri*, *L. plantarum*, and *L. salivarius*) and *Bifidobacterial* (*B. longum*), significantly reduced circulating cholesterol levels [25,143]. Oral administration of immobilized BSH from *L. buchneri* significantly reduced both triglycerides and serum cholesterol in a rodent model of hypercholesterolemia [144].

Liver cirrhosis in patients is associated with altered bile acid excretion that may lead to impaired small intestinal motility and mucosal congestion. *L. paracasei* N1115 reduces the translocation of *Veillonella* and *Streptococcus* to the intestine, improving liver function and inflammation in hepatobiliary disease patients [145]. Patients with liver cirrhosis have an abundance of *Streptococcus* and *Veillonella*, which are oral bacteria that invade the gut and contribute to cirrhotic deterioration. Translocation of this oral microbe is facilitated by altered bile acid secretion and reduction of gastric acid secretion [146]. In a placebo-controlled randomized study, the administration of a symbiotic capsule containing a BSH-active strain of *L. gasseri* with inulin resulted in significantly reduced total cholesterol and LDL compared to the placebo group [147]. Patients with hypercholesterolemia who consumed yogurt containing microencapsulated *L. reuteri* NCIMB 30242 and BSH-positive *L. reuteri* NCIMB 30242 consumption over 6 and 9 weeks had higher plasma deconjugated bile acid levels but lower total cholesterol, LDL, and non-HDL compared to the placebo group [148,149].

*L. reuteri* NCIMB 30242 was the first strain of probiotics to be marketed (Cardioviva^®^) for cholesterol-reducing purposes based on BSH and the bile salt deconjugation mechanism [90]. Yogurt containing *L. reuteri* CRL 1098 significantly decreased total circulating cholesterol and LDL in participants, while levels of HDL and triglycerides were unchanged [150]. Microbial BSH activity can impact lipid metabolism and reduce hypercholesterolemia, and most species of *Lactobacillus* that have a hypocholesterolemic effect in human trials have been patented [141]. This effect primarily relies on the ability of BSH bacteria to deconjugate primary BAs, thereby reducing cholesterol reabsorption [151].

*L. plantarum* KLDS 1.0344 supplementation may influence bile acid synthesis, as rats fed this probiotic with a high-cholesterol diet had increased Cyp7a1 and a concurrent decrease in cholesterol, ultimately excreting more fecal bile acids [152]. Treatment of HFD-fed mice with LGG inhibited *Fxr* and small heterodimer partner (*Shp*); both genes are involved in the transcriptional inhibition of *Cyp7a1*, and this disinhibition upregulates hepatic *Cyp7a1* expression. LGG treatment facilitates bile acid synthesis and lowers cholesterol [153]. Ref. [116] reported *L. paracasei* N1115 enhances *Cyp7a1* and *Ldl* receptor (LDLR) expression, leading to reduced LDL cholesterol that enhances bile acid metabolism [154]. found increased serum β-muricholic acid, a mouse-specific bile acid, with *L. gasseri* LA39 treatment in germ-free mice. Ref. [155] also linked increased tauro-β-muricholic acid levels with the abundance of the *Lactobacillus* genus in the GI tract. VSL#3 probiotics increased *Cyp7a1* and *Cy8b1* expression in mice [156]. The expression of additional hepatic bile acid synthesis genes *Cyp7b1, Cyp27a1, and Cyp8b1* was also significantly increased with *L. gasseri* LA39 treatment [154]. TGR5 activation by secondary BAs blocks the progression of hepatic inflammation and fibrosis. As *L. gasseri* LA39 produces secondary BA, it, therefore, may be a protective strategy [154]. Upregulation of *Cyp7a1* by *Lactobacillus* sp. enhances bile acid synthesis, lowers cholesterol levels and improves lipid metabolism. In addition to cholesterol-lowering effects, accumulating research has established the potential interplay between weight gain and obesity and gut microbiota-encoded BSHs through FXR signaling [141]. Studies of healthy fecal microbiota transplantation in patients with obesity resulted in a BA profile similar to that of the donor but did not reduce BMI [156]. Still, BSH enzymes represent a targeted strategy to control host lipidemia and protect against weight gain [141].

### 3.5. Lipids and Steatosis

Adipose and liver are the main tissues that mediate metabolism and lipogenesis; therefore, dysregulation of these organs is a potential contributor to metabolic disorders [120]. MASLD may encompass increased total cholesterol, triglycerides, and LDL cholesterol, as well as decreased HDL and gut microbiota dysbiosis induced by HFD. There have been several reported indications that lowering cholesterol, triglycerides, and LDL alleviates MASLD [157]. Dyslipidemia and elevated liver aminotransferases (AST and ALT) are markers of MASLD, and serum AST and ALT are used to characterize hepatic injury. Supplementation with three LAB strains (*Lactiplantibacillus plantarum* NCUH001046, *Limosilactobacillus reuteri* NCUH064003, and *Limosilactobacillus fermentum* NCUH003068) prevented body weight gain as well as accumulation of adipose and liver weight in obese mice. They also reduced serum cholesterol, triglycerides, and LDL cholesterol, while fecal cholesterol and triglyceride excretion were increased and showed significantly higher production of SCFAs, including butanoic acid, acetic acid, and propanoic acid. SCFAs activate AMPK signaling and induce GLP-1 secretion, which enhances the insulin receptor substrate and modulates lipid and glucose metabolism. It also facilitates β-oxidation of FFA via upregulation of the transcription factor peroxisomal proliferative activator receptor α (PPARα) [115]. *L. paracasei* LC-N1115 inhibits fat synthesis genes, including fatty acid synthase (*Fas*) and acetyl-CoA carboxylase (*Acc*) and reduces hepatic steatosis, body weight gain, white adipose mass and plasma cholesterol in HFD-fed mice [116]. *L. bulgaricus* supplementation in Western diet-induced MASLD mice alleviates hepatic lipid accumulation by downregulating NF-κB p65 expression, significantly preventing liver injury. Refs. [118,158] reported improved body and liver weight gain with LGG in diet-induced obese mice.

*L. kefiri* DH5 reduced hepatic steatosis, epididymal adiposity, and weight gain in HFD-fed mice; serum triglycerides and LDL cholesterol were also reduced via Pparα. These findings are consistent with [159] reporting *L. reuteri* and *L. acidophilus* significantly reduced cholesterol levels. This contrasts with [160], which demonstrated partial reduction of cholesterol with *L. casei* and *L. sakei* [161], highlighting important species and strain differences in *Lactobacillus* efficacy. Studies revealed that PPARα upregulates fatty acid oxidation to inhibit lipid accumulation, and *L. kefiri* DH5 increases adipose and hepatic PPARα expression, demonstrating anti-obesity effects [161]. *L. amylovorus* CP1563 improves lipid and glucose metabolism and reduces adiposity via upregulation of PPARα, and pre-and probiotics may function as effective PPARα agonists [162]. *L. kefiri* upregulates mRNA expression of lipid transport and oxidation genes in epididymal adipose tissue, which leads to catalysis of fatty acyl-CoA and suppression of lipogenesis, thus providing a mechanism for the lipolytic effects of *L. kefiri* [161].

Ref. [116] suggested that LC-N1115 given to HFD-fed mice suppresses cholesterol synthesis by increasing LDLR expression and decreasing LDL cholesterol levels. It also reduces body weight and white adipose and significantly lowers plasma cholesterol and triglyceride levels. Strains of *L. sakei* (OK67, ADM14, and WIKIM31) modulate lipid accumulation in adipose tissue, reduce serum triglyceride, inhibit lipogenesis genes (*Srebp1c*, *Cd36*, *Fas*, and *Cebpα*) and promote energy expenditure by increasing β-oxidation genes (*Cpt1α* and *Ucp2*) in adipose and liver [120]. Ref. [163] reported *L. plantarum* NA136 supplementation in HFD-fed mice decreased hepatic steatosis, decreased plasma lipids and LDL, and increased HDL. Administration of *L. plantarum* NA136 also significantly decreased circulating AST and ALT, an indication that L. plantarum NA136 attenuates hepatic injury and protects liver function.

AMPK maintains cellular energy homeostasis and controls glucose and lipid metabolism. It is activation arises by cellular starvation due to *an* increased ratio of intracellular AMP to ATP; its activation also regulates fatty acid oxidation, switching off anabolic pathways by phosphorylating many downstream substrates involved in glycolysis, glucose metabolism and mitochondria function [164]. Acetyl-CoA carboxylase (ACC) and fatty acid synthase (FAS) promote lipogenesis and malonyl-CoA availability for fatty acid synthesis in hepatocytes, thus contributing to steatosis, dyslipidemia, and diabetes. [165] AMPK phosphorylation of ACC reduces its activity to inhibit de novo lipogenesis and activate fatty acid oxidation [166]. *L. plantarum* NA136 increased AMPK phosphorylation, leading to increased ACC phosphorylation inhibited de novo lipogenesis [163]. Refs. [163,167] further suggested that the decreased cholesterol and FFA seen in L. plantarum NA136- and HFD-fed mice is due to decreased Srebp-1 and Fas levels. *L. acidophilus* (La5) and Bifidobacterium lactis given to people older than 55 years improved serum total cholesterol, triglycerides, HDL and LDL compared to placebo [168]. *L. acidophilus* La5 and *Bifidobacterium lactis* Bb1 for eight weeks improved hepatic ALT, AST, LDL cholesterol, and serum total cholesterol in patients with MASLD in a double-masked, randomized, controlled trial. However, no significant differences were observed in serum triglycerides, glucose or HDL levels [157]. Obese children with MASLD received *L. acidophilus*, *B. lactis*, *B. bifidum*, and *L. rhamnosus* or placebo for 12 weeks. This intervention significantly reduced serum cholesterol, triglycerides, LDL and AST compared to the placebo group [169]. Obese children with sonographic MASLD treated with probiotic capsule (containing Lactobacillus acidophilus ATCC B3208, *Bifidobacterium lactis* DSMZ 32269, Bifidobacterium bifidum ATCC SD6576, and *Lactobacillus rhamnosus* DSMZ 21690) exhibited reduced ALT, AST, total cholesterol, LDL-C, and TG in serum [169]. In one study, patients with MASLD were given daily Lactobacillus bulgaricus and Streptococcus thermophilus, which resulted in decreased ALT, AST and GGT [170]. Patients with T2DM and MASLD were given live multi-strain supplements of 14 probiotic bacteria genera (including Lactobacillus, Lactococcus, Bifidobacterium, Propionibacterium, and Acetobacter) had deceased AST, GGT, TNF-α and IL-6 [171]. In a randomized double-blind controlled trial, patients with MASLD receiving daily probiotic yogurt containing *Lactobacillus acidophilus* La5 and Bifidobacterium lactis Bb12 for 6 wk had reduced total cholesterol and LDL-C compared with the control group [172]. *Lactobacillus casei* 01 intervention group serum fetuin-A level, fasting blood sugar, insulin concentration, and insulin resistance were significantly decreased, and serum sirtuin1 (*SIRT1*) level was significantly increased compared to the placebo group. Administration of heat-treated Lactobacillus plantarum OLL2712 (OLL2712) cells improved glucose and lipid metabolism by suppressing chronic inflammation in mouse models. Finally, a preliminary clinical study found that ingestion of heat-treated OLL2712 cells in overweight, healthy adults reduced body fat accumulation and prevented the deterioration of glycemic control and chronic inflammation [173]. Generally, specific strains of *Lactobacillus* species may be a useful treatment strategy for liver steatosis and hyperlipidemia, including MASLD and MASH.

### 3.6. Inflammation and Fibrosis

Chronic systemic inflammation is a symptom of MASLD. Recent studies demonstrated that systemic LPS is due to increased intestinal permeability and that activation of TLR4 signaling triggers the secretion of proinflammatory cytokines through NF-κB signaling [174].

Ref. [115] reported that LPS positively correlated with gut permeability and inflammatory cytokine expression. *Lactiplantibacillus plantarum* NCUH001046 was given to obese mice. Chronic inflammation induced by long-term HFD was attenuated with a reduction in TNF-α while the anti-inflammatory cytokine IL-10 was increased. Several studies have also reported that reduced inflammatory cytokines correlate with improved glucose and lipid metabolism. Leptin and adiponectin are major adipokines that alleviate obesity. Leptin increases FFA oxidation and prevents liver lipogenesis, while adiponectin reduces insulin resistance and metabolic disorders; enhanced signaling of leptin and adiponectin also suppress inflammation. Three LABs (*Lactiplantibacillus plantarum*, *Limosilactobacillus reuteri*, and *Limosilactobacillus fermentum*), given to high-fat-diet-induced obese mice, significantly elevated serum adiponectin and leptin through activation AMPK signaling [115]. *L. paracasei* N1115 (LC-N1115) downregulated *TLR4* and *IL-1β* mRNA expression in adipose tissue [116]. Elevated *Il-1β*, *Il-6*, and *Tnf-α* induced by a long-term Western diet were significantly reduced by *Lactobacillus* (*L. bulgaricus*, *L. casei*, *L. delbrueckii*, *L. acidophilus*) in mice [118].

In another study, patients with MASLD received a multi-probiotic intervention of mixed *Acetobacter*, *Bifidobacterium*, *Propionibacterium*, *Lactococcus*, *Lactobacillus* or placebo for 8 weeks, and this significantly improved fatty liver index, AST, TNFα, and IL-6 compared to placebo [175]. 10-week treatment of VSL#3 to patients with MASLD, primarily consisting of *Lactobacillus* and *Bifidobacterium* genera, significantly decreased interferon-γ and TNF-α in a double-blind study [127]. This finding is significant as it suggests a potential therapeutic role of VSL#3 probiotics in managing MASLD. The treatment also led to improved insulin sensitivity and serum glucose, total cholesterol, triglycerides, LDL, and HDL, reduced IL-1β, IL-6 and TNF-α, and a decrease in markers of oxidative stress as well as an improvement in hepatic fat [176,177]. *Lactobacillus pantarum* produces lipoteichoic acid, which elicits an anti-inflammatory response in both human intestinal epithelial cells via blockage of inflammatory IL-8 [178]. Multi-strain probiotics of Lactobacillus and Bifidobacterium spp resulted in an improvement in major histological parameters of NAFLD/MASLD as well as in decreases in cytokine levels and serum ALT [179]. These findings suggest that human trials involving probiotic supplementation to alleviate MASLD should consider several key factors, such as the timing of probiotic supplementation, the stage of liver disease and even the use of probiotics before disease onset [180].

## 4. Discussion

Weight loss and diet modification are recommended therapies for MASLD, and the novel drug Resmetirom was approved this year to treat MASH. However, its effectiveness is limited, and more therapies are still needed. MASLD is ingrained in complex pathophysiological mechanisms of metabolic syndrome, including obesity, T2DM, dyslipidemia, and insulin resistance, and it is crucial to develop more effective therapies for this complex condition. Drugs that target the microbiome, like probiotics, prebiotics, synbiotics, or fecal microbiota transplant (FMT), represent several novel avenues to tackle this disorder [181]. Beneficial effects of probiotics have been demonstrated in in vivo and in vitro studies, with *Lactobacillus* and *Bifidobacterium* being the most widely used genera. Lactobacillus species play a crucial role in intestinal microbiota restoration that improves barrier integrity. Its adherence to the intestinal mucosa controls the permeability of other bacteria, nutrients from food, and molecules resulting from cellular metabolism. It produces SCFCAs that are crucial to liver function. They also decrease blood lipid levels and hepatic enzymes and reduce hepatic fat accumulation and inflammation, thereby mitigating liver pathology [182]. Here, we reviewed various strains of *Lactobacillus* that improve intestinal barrier function and enhance tight junction proteins to maintain intestinal barrier integrity. These species also modulate the immune response by promoting anti-inflammatory cytokines and reducing pro-inflammatory cytokines, producing SCFAS that nourish the intestinal lining, enhancing barrier function, and secreting antimicrobial peptides that inhibit the growth of pathogenic bacteria [183]. Probiotics can modulate tight junction proteins by stimulating TLRs that stimulate the intestinal epithelium and enhance these proteins’ expression, thus maintaining the integrity of the intestinal barrier [184]. A functional intestinal barrier prevents the translocation of LPS and other microbial products to the systemic bloodstream. This helps to reduce the activation of immune cells in the lamina propria, preventing the release of proinflammatory cytokines. Finally, maintenance of the intestinal barrier prevents LPS activation of the TL4 pathway, thereby reducing intestinal and hepatic inflammation [11]. While evidence demonstrates that *Lactobacillus* positively contributes to antimicrobial activity, microbiota modulation, and immunomodulatory effects in MASLD, most of the mechanisms of action of these probiotics have not been fully clarified. A major challenge lies in delineating the complex signaling relationships between bacteria and host tissues, as well as inter-species interactions, many of which are strain-specific. Future research focused on elucidating the mechanisms of action of Lactobacillus and other probiotics will give much-needed insight not only into their viability as a treatment for MASLD and MASH but also for many other metabolic diseases.

### Limitations of Probiotic Therapy and the Challenge of Sustaining Therapeutic Levels of Probiotics in the Gut

Many probiotic strains are derived from species with a long history of safe use in foods or from microorganisms that colonize healthy gastrointestinal tracts (*acidophilus*, *casei, fermentum*, *gasseri*, *johnsonii, paracasei*, *plantarum*, *rhamnosus*, and *salivarius* from *Lactobacillus* and *adolescents*, *animals*, *bifidum*, *breve*, and *long* from Bifidobacterium). Most strains of *Lactobacillus* are not pathogenic and are part of the human flora, but some reported cases of probiotics use have been linked to bacteremia or fungemia infections in individuals who were severely ill or immunocompromised [185]. The systematic review of efficacy and safety of probiotics in MASLD treatment was analyzed in 21 studies involving 1037 participants after the probiotic intervention. This review found significant improvement in liver function (ALT, AST, GGT), improved steatosis, and reduced blood sugar, while no severe adverse reactions were reported [186]. Because probiotics can interact with commensal bacteria and immediately affect the host, especially in vulnerable patients, it is necessary to understand the methods of action of the specific strain, clarify the strain benefit, and define the consumption levels required. To develop effective therapy against side effects, long-term clinical and mechanistic investigations are necessary to understand better the interface between bacteria, host cells, mucus, and immune systems. Because the gut microbiome is unique, individual responses to dietary changes can vary. The gut microbiota is affected by dietary habits, and studies in different regions may lead to biases in the biological characteristics of the gut microbiota [186]. Reports of interactions of probiotics with known drugs are also scarce, though probiotics may also affect the bioavailability, efficacy, and safety of drugs [187], and probiotics are regulated less stringently than medical and pharmaceutical products [188]. The diversity of strain dependence, in conjunction with dosage and lifestyle, influences the efficacy of clinical trials. Nevertheless, ref. [189] reported *L*. *acidophilus* L-92 mediated immune modulation via surface layer protein slpA, *L*. *rhamnosus* mediated modulation of TNF-α, IL-6/10/12 in the intestinal mucous via cell surface appendage, and that *B*. *longum* stimulated IL-10 secretion and modulation of proinflammatory cytokine and helper-T cells. Overall, *Lactobacillus* and *Bifidobacterium* have distinct health benefits ranging from immune system fortification to potential anti-cancer properties, but well-designed studies are still required to identify mechanisms of action and predict adverse effects.

## 5. Conclusions

From the gut microbiota to energy homeostasis, glucose metabolism, and lipid metabolism of the host, the use of probiotics has emerged as a strategy for preventing and treating MASLD. At the same time, the potential of probiotics to modulate the composition of the gut microbiota and inflammatory pathways via the gut-liver axis to attenuate MASLD and metabolic disorders is understudied and largely unknown. Here, *Lactobacillus* species were identified as promising probiotics to ameliorate MASLD due to their beneficial effects on lipid and bile acid metabolism, inflammation and intestinal barrier function.

## Data Availability

No new data were created or analyzed in this study. Data sharing is not applicable to this article.

## References

[1] Loomba R., Sanyal A.J. (2013). The global NAFLD epidemic. Nat. Rev. Gastroenterol. Hepatol..

[2] Meyer L.F., Musante C.J., Allen R. (2023). A continuous-time Markov chain model of fibrosis progression in NAFLD and NASH. Front. Med..

[3] Loomba R., Friedman S.L., Shulman G.I. (2021). Mechanisms and disease consequences of nonalcoholic fatty liver disease. Cell.

[4] Basu R., Noureddin M., Clark J.M. (2022). Nonalcoholic Fatty Liver Disease: Review of Management for Primary Care Providers. Mayo Clin. Proc..

[5] Buzzetti E., Pinzani M., Tsochatzis E.A. (2016). The multiple-hit pathogenesis of non-alcoholic fatty liver disease (NAFLD). Metabolism.

[6] Ferenc K., Sokal-Dembowska A., Helma K., Motyka E., Jarmakiewicz-Czaja S., Filip R. (2024). Modulation of the Gut Microbiota by Nutrition and Its Relationship to Epigenetics. Int. J. Mol. Sci..

[7] Wong V.W.-S., Ekstedt M., Wong G.L.-H., Hagström H. (2023). Changing epidemiology, global trends and implications for outcomes of NAFLD. J. Hepatol..

[8] Beiriger J., Chauhan K., Khan A., Shahzad T., Parra N.S., Zhang P., Chen S., Nguyen A., Yan B., Bruckbauer J. (2023). Advancements in Understanding and Treating NAFLD: A Comprehensive Review of Metabolic-Associated Fatty Liver Disease and Emerging Therapies. Livers.

[9] Pierantonelli I., Svegliati-Baroni G. (2019). Nonalcoholic Fatty Liver Disease: Basic Pathogenetic Mechanisms in the Progression From NAFLD to NASH. Transplantation.

[10] Kawano Y., Cohen D.E. (2013). Mechanisms of hepatic triglyceride accumulation in non-alcoholic fatty liver disease. J. Gastroenterol..

[11] Guo X., Yin X., Liu Z., Wang J. (2022). Non-Alcoholic Fatty Liver Disease (NAFLD) Pathogenesis and Natural Products for Prevention and Treatment. Int. J. Mol. Sci..

[12] Bessone F., Razori M.V., Roma M.G. (2019). Molecular pathways of nonalcoholic fatty liver disease development and progression. Cell. Mol. Life Sci..

[13] Jandhyala S.M., Talukdar R., Subramanyam C., Vuyyuru H., Sasikala M., Nageshwar Reddy D. (2015). Role of the normal gut microbiota. World J. Gastroenterol..

[14] Rastelli M., Cani P.D., Knauf C. (2019). The Gut Microbiome Influences Host Endocrine Functions. Endocr. Rev..

[15] Hrncir T., Hrncirova L., Kverka M., Hromadka R., Machova V., Trckova E., Kostovcikova K., Kralickova P., Krejsek J., Tlaskalova-Hogenova H. (2021). Gut Microbiota and NAFLD: Pathogenetic Mechanisms, Microbiota Signatures, and Therapeutic Interventions. Microorganisms.

[16] Jasirwan C.O.M., Lesmana C.R.A., Hasan I., Sulaiman A.S., Gani R.A. (2019). The role of gut microbiota in non-alcoholic fatty liver disease: Pathways of mechanisms. Biosci. Microbiota Food Heal..

[17] Carpino G., Del Ben M., Pastori D., Carnevale R., Baratta F., Overi D., Francis H., Cardinale V., Onori P., Safarikia S. (2020). Increased Liver Localization of Lipopolysaccharides in Human and Experimental NAFLD. Hepatology.

[18] An L., Wirth U., Koch D., Schirren M., Drefs M., Koliogiannis D., Nieß H., Andrassy J., Guba M., Bazhin A.V. (2022). The Role of Gut-Derived Lipopolysaccharides and the Intestinal Barrier in Fatty Liver Diseases. J. Gastrointest. Surg..

[19] Liu L., Yin M., Gao J., Yu C., Lin J., Wu A., Zhu J., Xu C., Liu X. (2022). Intestinal Barrier Function in the Pathogenesis of Nonalcoholic Fatty Liver Disease. J. Clin. Transl. Hepatol..

[20] Di Tommaso N., Gasbarrini A., Ponziani F.R. (2021). Intestinal Barrier in Human Health and Disease. Int. J. Environ. Res. Public Heal..

[21] Nicoletti A., Ponziani F.R., Biolato M., Valenza V., Marrone G., Sganga G., Gasbarrini A., Miele L., Grieco A. (2019). Intestinal permeability in the pathogenesis of liver damage: From non-alcoholic fatty liver disease to liver transplantation. World J. Gastroenterol..

[22] Cornick S., Tawiah A., Chadee K. (2015). Roles and regulation of the mucus barrier in the gut. Tissue Barriers.

[23] Hartmann P., Seebauer C.T., Mazagova M., Horvath A., Wang L., Llorente C., Varki N.M., Brandl K., Ho S.B., Schnabl B. (2016). Deficiency of intestinal mucin-2 protects mice from diet-induced fatty liver disease and obesity. Am. J. Physiol. Liver Physiol..

[24] Johansson M.E.V., Larsson J.M.H., Hansson G.C. (2011). The two mucus layers of colon are organized by the MUC2 mucin, whereas the outer layer is a legislator of host–microbial interactions. Proc. Natl. Acad. Sci. USA.

[25] Wang G., Huang W., Xia Y., Xiong Z., Ai L. (2019). Cholesterol-lowering potentials of *Lactobacillus* strain overexpression of bile salt hydrolase on high cholesterol diet-induced hypercholesterolemic mice. Food Funct..

[26] Seki E., Brenner D.A. (2008). Toll-like receptors and adaptor molecules in liver disease. Hepatology.

[27] Ilan Y. (2016). Review article: Novel methods for the treatment of non-alcoholic steatohepatitis—targeting the gut immune system to decrease the systemic inflammatory response without immune suppression. Aliment. Pharmacol. Ther..

[28] Takiishi T., Fenero C.I.M., Câmara N.O.S. (2017). Intestinal barrier and gut microbiota: Shaping our immune responses throughout life. Tissue Barriers.

[29] Chakaroun R.M., Massier L., Kovacs P. (2020). Gut Microbiome, Intestinal Permeability, and Tissue Bacteria in Metabolic Disease: Perpetrators or Bystanders?. Nutrients.

[30] Cui Y., Wang Q., Chang R., Zhou X., Xu C. (2019). Intestinal Barrier Function-Non-alcoholic Fatty Liver Disease Interactions and Possible Role of Gut Microbiota. J. Agric. Food. Chem..

[31] Xin D., Zong-Shun L., Bang-Mao W., Lu Z. (2014). Expression of intestinal tight junction proteins in patients with non-alcoholic fatty liver disease. Hepatogastroenterology.

[32] Rahman K., Desai C., Iyer S.S., Thorn N.E., Kumar P., Liu Y., Smith T., Neish A.S., Li H., Tan S. (2016). Loss of Junctional Adhesion Molecule A Promotes Severe Steatohepatitis in Mice on a Diet High in Saturated Fat, Fructose, and Cholesterol. Gastroenterology.

[33] Allam-Ndoul B., Castonguay-Paradis S., Veilleux A. (2020). Gut Microbiota and Intestinal Trans-Epithelial Permeability. Int. J. Mol. Sci..

[34] Miele L., Valenza V., La Torre G., Montalto M., Cammarota G., Ricci R., Mascianà R., Forgione A., Gabrieli M.L., Perotti G. (2009). Increased intestinal permeability and tight junction alterations in nonalcoholic fatty liver disease. Hepatology.

[35] Gäbele E., Dostert K., Hofmann C., Wiest R., Schölmerich J., Hellerbrand C., Obermeier F. (2011). DSS induced colitis increases portal LPS levels and enhances hepatic inflammation and fibrogenesis in experimental NASH. J. Hepatol..

[36] Albillos A., de Gottardi A., Rescigno M. (2020). The gut-liver axis in liver disease: Pathophysiological basis for therapy. J. Hepatol..

[37] Potts R.A., Tiffany C.M., Pakpour N., Lokken K.L., Tiffany C.R., Cheung K., Tsolis R.M., Luckhart S. (2016). Mast cells and histamine alter intestinal permeability during malaria parasite infection. Immunobiology.

[38] Konturek P.C., Harsch I.A., Konturek K., Schink M., Konturek T., Neurath M.F., Zopf Y. (2018). Gut–Liver Axis: How Do Gut Bacteria Influence the Liver?. Med. Sci..

[39] Henao-Mejia J., Elinav E., Jin C., Hao L., Mehal W.Z., Strowig T., Thaiss C.A., Kau A.L., Eisenbarth S.C., Jurczak M.J. (2012). Inflammasome-mediated dysbiosis regulates progression of NAFLD and obesity. Nature.

[40] Mei W., Hao Y., Xie H., Ni Y., Zhao R. (2020). Hepatic Inflammatory Response to Exogenous LPS Challenge is Exacerbated in Broilers with Fatty Liver Disease. Animals.

[41] Kuwabara W.M.T., Yokota C.N.F., Curi R., Alba-Loureiro T.C. (2018). Obesity and Type 2 Diabetes mellitus induce lipopolysaccharide tolerance in rat neutrophils. Sci. Rep..

[42] A Hegazy M., Mogawer S.M., Alnaggar A.R.L.R., A Ghoniem O., Samie R.M.A. (2020). Serum LPS and CD163 Biomarkers Confirming the Role of Gut Dysbiosis in Overweight Patients with NASH. Diabetes Metab. Syndr. Obes. Targets Ther..

[43] Barchetta I., Cimini F.A., Sentinelli F., Chiappetta C., Di Cristofano C., Silecchia G., Leonetti F., Baroni M.G., Cavallo M.G. (2023). Reduced Lipopolysaccharide-Binding Protein (LBP) Levels Are Associated with Non-Alcoholic Fatty Liver Disease (NAFLD) and Adipose Inflammation in Human Obesity. Int. J. Mol. Sci..

[44] Kolodziejczyk A.A., Zheng D., Shibolet O., Elinav E. (2019). The role of the microbiome in NAFLD and NASH. EMBO Mol. Med..

[45] Xue L., He J., Gao N., Lu X., Li M., Wu X., Liu Z., Jin Y., Liu J., Xu J. (2017). Probiotics may delay the progression of nonalcoholic fatty liver disease by restoring the gut microbiota structure and improving intestinal endotoxemia. Sci. Rep..

[46] Tack C.J., Stienstra R., Joosten L.A.B., Netea M.G. (2012). Inflammation links excess fat to insulin resistance: The role of the interleukin-1 family. Immunol. Rev..

[47] Plomgaard P., Bouzakri K., Krogh-Madsen R., Mittendorfer B., Zierath J.R., Pedersen B.K. (2005). Tumor Necrosis Factor-α Induces Skeletal Muscle Insulin Resistance in Healthy Human Subjects via Inhibition of Akt Substrate 160 Phosphorylation. Diabetes.

[48] Maestri M., Santopaolo F., Pompili M., Gasbarrini A., Ponziani F.R. (2023). Gut microbiota modulation in patients with non-alcoholic fatty liver disease: Effects of current treatments and future strategies. Front. Nutr..

[49] Ji Y., Yin Y., Li Z., Zhang W. (2019). Gut Microbiota-Derived Components and Metabolites in the Progression of Non-Alcoholic Fatty Liver Disease (NAFLD). Nutrients.

[50] Xia Y., Ren M., Yang J., Cai C., Cheng W., Zhou X., Lu D., Ji F. (2022). Gut microbiome and microbial metabolites in NAFLD and after bariatric surgery: Correlation and causality. Front. Microbiol..

[51] Soliman M.M., Ahmed M.M., Salah-Eldin A.-E., Abdel-Aal A.A.-A. (2011). Butyrate regulates leptin expression through different signaling pathways in adipocytes. J. Vet. Sci..

[52] Perry R.J., Peng L., Barry N.A., Cline G.W., Zhang D., Cardone R.L., Petersen K.F., Kibbey R.G., Goodman A.L., Shulman G.I. (2016). Acetate mediates a microbiome–brain–β-cell axis to promote metabolic syndrome. Nature.

[53] Hernández M.A.G., Canfora E.E., Jocken J.W.E., Blaak E.E. (2019). The Short-Chain Fatty Acid Acetate in Body Weight Control and Insulin Sensitivity. Nutrients.

[54] Karaki S.-I., Tazoe H., Hayashi H., Kashiwabara H., Tooyama K., Suzuki Y., Kuwahara A. (2008). Expression of the short-chain fatty acid receptor, GPR43, in the human colon. J. Mol. Histol..

[55] Tazoe H., Otomo Y., Karaki S.-I., Kato I., Fukami Y., Terasaki M., Kuwahara A. (2009). Expression of short-chain fatty acid receptor GPR41 in the human colon. Biomed. Res..

[56] Nøhr M.K., Pedersen M.H., Gille A., Egerod K.L., Engelstoft M.S., Husted A.S., Sichlau R.M., Grunddal K.V., Seier Poulsen S., Han S. (2013). GPR41/FFAR3 and GPR43/FFAR2 as Cosensors for Short-Chain Fatty Acids in Enteroendocrine Cells vs FFAR3 in Enteric Neurons and FFAR2 in Enteric Leukocytes. Endocrinology.

[57] Maruta H., Yoshimura Y., Araki A., Kimoto M., Takahashi Y., Yamashita H. (2016). Activation of AMP-Activated Protein Kinase and Stimulation of Energy Metabolism by Acetic Acid in L6 Myotube Cells. PLoS ONE.

[58] Park J.W., Kim H.Y., Kim M.G., Jeong S., Yun C.-H., Han S.H. (2019). Short-chain Fatty Acids Inhibit Staphylococcal Lipoprotein-induced Nitric Oxide Production in Murine Macrophages. Immune Netw..

[59] Juanola O., Ferrusquía-Acosta J., García-Villalba R., Zapater P., Magaz M., Marín A., Olivas P., Baiges A., Bellot P., Turon F. (2019). Circulating levels of butyrate are inversely related to portal hypertension, endotoxemia, and systemic inflammation in patients with cirrhosis. FASEB J..

[60] de la Cuesta-Zuluaga J., Mueller N.T., Corrales-Agudelo V., Velásquez-Mejía E.P., Carmona J.A., Abad J.M., Escobar J.S. (2017). Metformin Is Associated With Higher Relative Abundance of Mucin-Degrading Akkermansia muciniphilaand Several Short-Chain Fatty Acid–Producing Microbiota in the Gut. Diabetes Care.

[61] Xiong J., Chen X., Zhao Z., Liao Y., Zhou T., Xiang Q. (2022). A potential link between plasma short-chain fatty acids, TNF-α level and disease progression in non-alcoholic fatty liver disease: A retrospective study. Exp. Ther. Med..

[62] Behary J., Amorim N., Jiang X.-T., Raposo A., Gong L., McGovern E., Ibrahim R., Chu F., Stephens C., Jebeili H. (2021). Gut microbiota impact on the peripheral immune response in non-alcoholic fatty liver disease related hepatocellular carcinoma. Nat. Commun..

[63] Tsai H.-J., Hung W.-C., Hung W.-W., Lee Y.-J., Chen Y.-C., Lee C.-Y., Tsai Y.-C., Dai C.-Y. (2023). Circulating Short-Chain Fatty Acids and Non-Alcoholic Fatty Liver Disease Severity in Patients with Type 2 Diabetes Mellitus. Nutrients.

[64] Valdes A.M., Walter J., Segal E., Spector T.D. (2018). Role of the gut microbiota in nutrition and health. BMJ.

[65] Waseem M.R., Shin A., Siwiec R., James-Stevenson T., Bohm M., Rogers N., Wo J., Waseem L., Gupta A., Jarrett M. (2023). Associations of Fecal Short Chain Fatty Acids With Colonic Transit, Fecal Bile Acid, and Food Intake in Irritable Bowel Syndrome. Clin. Transl. Gastroenterol..

[66] Facchin S., Bertin L., Bonazzi E., Lorenzon G., De Barba C., Barberio B., Zingone F., Maniero D., Scarpa M., Ruffolo C. (2024). Short-Chain Fatty Acids and Human Health: From Metabolic Pathways to Current Therapeutic Implications. Life.

[67] Thing M., Werge M.P., Kimer N., Hetland L.E., Rashu E.B., Nabilou P., Junker A.E., Galsgaard E.D., Bendtsen F., Laupsa-Borge J. (2024). Targeted metabolomics reveals plasma short-chain fatty acids are associated with metabolic dysfunction-associated steatotic liver disease. BMC Gastroenterol..

[68] Zhang X., Coker O.O., Chu E.S., Fu K., Lau H.C.H., Wang Y.-X., Chan A.W.H., Wei H., Yang X., Sung J.J.Y. (2021). Dietary cholesterol drives fatty liver-associated liver cancer by modulating gut microbiota and metabolites. Gut.

[69] Dangana E., Omolekulo T., Areola E., Olaniyi K., Soladoye A., Olatunji L. (2020). Sodium acetate protects against nicotine-induced excess hepatic lipid in male rats by suppressing xanthine oxidase activity. Chem. Interact..

[70] Zhou D., Fan J.-G. (2019). Microbial metabolites in non-alcoholic fatty liver disease. World J. Gastroenterol..

[71] Zhou D., Pan Q., Xin F.-Z., Zhang R.-N., He C.-X., Chen G.-Y., Liu C., Chen Y.-W., Fan J.-G. (2017). Sodium butyrate attenuates high-fat diet-induced steatohepatitis in mice by improving gut microbiota and gastrointestinal barrier. World J. Gastroenterol..

[72] Zhao Z.-H., Wang Z.-X., Zhou D., Han Y., Ma F., Hu Z., Xin F.-Z., Liu X.-L., Ren T.-Y., Zhang F. (2021). Sodium Butyrate Supplementation Inhibits Hepatic Steatosis by Stimulating Liver Kinase B1 and Insulin-Induced Gene. Cell. Mol. Gastroenterol. Hepatol..

[73] Di Ciaula A., Baj J., Garruti G., Celano G., De Angelis M., Wang H.H., Di Palo D.M., Bonfrate L., Wang D.Q.-H., Portincasa P. (2020). Liver Steatosis, Gut-Liver Axis, Microbiome and Environmental Factors. A Never-Ending Bidirectional Cross-Talk. J. Clin. Med..

[74] Larabi A.B., Masson H.L.P., Bäumler A.J. (2023). Bile acids as modulators of gut microbiota composition and function. Gut Microbes.

[75] Chiang J.Y.L., Ferrell J.M. (2018). Bile Acid Metabolism in Liver Pathobiology. Gene Expr..

[76] Nishida A., Inoue R., Inatomi O., Bamba S., Naito Y., Andoh A. (2018). Gut microbiota in the pathogenesis of inflammatory bowel disease. Clin. J. Gastroenterol..

[77] Foley M.H., Walker M.E., Stewart A.K., O’flaherty S., Gentry E.C., Patel S., Beaty V.V., Allen G., Pan M., Simpson J.B. (2023). Bile salt hydrolases shape the bile acid landscape and restrict Clostridioides difficile growth in the murine gut. Nat. Microbiol..

[78] Gillard J., Clerbaux L.-A., Nachit M., Sempoux C., Staels B., Bindels L.B., Tailleux A., Leclercq I.A. (2022). Bile acids contribute to the development of non-alcoholic steatohepatitis in mice. JHEP Rep..

[79] Guan B., Tong J., Hao H., Yang Z., Chen K., Xu H., Wang A. (2022). Bile acid coordinates microbiota homeostasis and systemic immunometabolism in cardiometabolic diseases. Acta Pharm. Sin. B.

[80] Cruz-Ramón V., Chinchilla-López P., Ramírez-Pérez O., Méndez-Sánchez N. (2017). Bile Acids in Nonalcoholic Fatty Liver Disease: New Concepts and Therapeutic Advances. Ann. Hepatol..

[81] Lake A.D., Novak P., Shipkova P., Aranibar N., Robertson D., Reily M.D., Lu Z., Lehman-McKeeman L.D., Cherrington N.J. (2013). Decreased hepatotoxic bile acid composition and altered synthesis in progressive human nonalcoholic fatty liver disease. Toxicol. Appl. Pharmacol..

[82] Gottlieb A., Canbay A., Gottlieb A., Canbay A. (2019). Why Bile Acids Are So Important in Non-Alcoholic Fatty Liver Disease (NAFLD) Progression. Cells.

[83] Ferrell J.M., Chiang J.Y. (2021). Bile acid receptors and signaling crosstalk in the liver, gut and brain. Liver Res..

[84] Yang Z.-X., Shen W., Sun H. (2010). Effects of nuclear receptor FXR on the regulation of liver lipid metabolism in patients with non-alcoholic fatty liver disease. Hepatol. Int..

[85] Chiang J.Y.L., Ferrell J.M. (2019). Bile Acids as Metabolic Regulators and Nutrient Sensors. Annu. Rev. Nutr..

[86] Lai J., Luo L., Zhou T., Feng X., Ye J., Zhong B. (2023). Alterations in Circulating Bile Acids in Metabolic Dysfunction-Associated Steatotic Liver Disease: A Systematic Review and Meta-Analysis. Biomolecules.

[87] Bilson J., Scorletti E., Swann J.R., Byrne C.D. (2024). Bile Acids as Emerging Players at the Intersection of Steatotic Liver Disease and Cardiovascular Diseases. Biomolecules.

[88] Zou Y., Ju X., Chen W., Yuan J., Wang Z., Aluko R.E., He R. (2020). Rice bran attenuated obesity *via* alleviating dyslipidemia, browning of white adipocytes and modulating gut microbiota in high-fat diet-induced obese mice. Food Funct..

[89] Turnbaugh P.J., Hamady M., Yatsunenko T., Cantarel B.L., Duncan A., Ley R.E., Sogin M.L., Jones W.J., Roe B.A., Affourtit J.P. (2009). A core gut microbiome in obese and lean twins. Nature.

[90] Michail S., Lin M., Frey M.R., Fanter R., Paliy O., Hilbush B., Reo N.V. (2015). Altered gut microbial energy and metabolism in children with non-alcoholic fatty liver disease. FEMS Microbiol. Ecol..

[91] Ley R.E., Turnbaugh P.J., Klein S., Gordon J.I. (2006). Human gut microbes associated with obesity. Nature.

[92] Chan Y.K., Estaki M., Gibson D.L. (2013). Clinical Consequences of Diet-Induced Dysbiosis. Ann. Nutr. Metab..

[93] Yuan J., Zhang J., Luo Q., Peng L. (2024). Effects of nonalcoholic fatty liver disease on sarcopenia: Evidence from genetic methods. Sci. Rep..

[94] Jiang C., Wang Y., Fu W., Zhang G., Feng X., Wang X., Wang F., Zhang L., Deng Y. (2022). Association between sarcopenia and prognosis of hepatocellular carcinoma: A systematic review and meta-analysis. Front. Nutr..

[95] Shokri-Mashhadi N., Navab F., Ansari S., Rouhani M.H., Hajhashemy Z., Saraf-Bank S. (2023). A meta-analysis of the effect of probiotic administration on age-related sarcopenia. Food Sci. Nutr..

[96] Baek J.-S., Shin Y.-J., Ma X., Park H.-S., Hwang Y.-H., Kim D.-H. (2023). Bifidobacterium bifidum and Lactobacillus paracasei alleviate sarcopenia and cognitive impairment in aged mice by regulating gut microbiota-mediated AKT, NF-κB, and FOXO3a signaling pathways. Immun. Ageing.

[97] Chen L., Chang S., Chang H., Wu C., Pan C., Chang C., Chan C., Huang H. (2022). Probiotic supplementation attenuates age-related sarcopenia via the gut–muscle axis in SAMP8 mice. J. Cachexia Sarcopenia Muscle.

[98] Hemarajata P., Versalovic J. (2013). Effects of probiotics on gut microbiota: Mechanisms of intestinal immunomodulation and neuromodulation. Ther. Adv. Gastroenterol..

[99] Afzaal M., Saeed F., Shah Y.A., Hussain M., Rabail R., Socol C.T., Hassoun A., Pateiro M., Lorenzo J.M., Rusu A.V. (2022). Human gut microbiota in health and disease: Unveiling the relationship. Front. Microbiol..

[100] Karlsson F.H., Nookaew I., Petranovic D., Nielsen J. (2011). Prospects for systems biology and modeling of the gut microbiome. Trends Biotechnol..

[101] Skoufou M., Tsigalou C., Vradelis S., Bezirtzoglou E. (2024). The Networked Interaction between Probiotics and Intestine in Health and Disease: A Promising Success Story. Microorganisms.

[102] Gratz S.W. (2010). Probiotics and gut health: A special focus on liver diseases. World J. Gastroenterol..

[103] Dempsey E., Corr S.C. (2022). Lactobacillus spp. for Gastrointestinal Health: Current and Future Perspectives. Front. Immunol..

[104] Żukiewicz-Sobczak W., Wróblewska P., Adamczuk P., Silny W. (2014). Probiotic lactic acid bacteria and their potential in the prevention and treatment of allergic diseases. Central Eur. J. Immunol..

[105] Di Cerbo A., Palmieri B., Aponte M., Morales-Medina J.C., Iannitti T. (2016). Mechanisms and therapeutic effectiveness of lactobacilli. J. Clin. Pathol..

[106] Latif A., Shehzad A., Niazi S., Zahid A., Ashraf W., Iqbal M.W., Rehman A., Riaz T., Aadil R.M., Khan I.M. (2023). Probiotics: Mechanism of action, health benefits and their application in food industries. Front. Microbiol..

[107] Kanmani P., Kim H. (2022). Probiotics counteract the expression of hepatic profibrotic genes via the attenuation of TGF-β/SMAD signaling and autophagy in hepatic stellate cells. PLoS ONE.

[108] Khushboo, Karnwal A., Malik T. (2023). Characterization and selection of probiotic lactic acid bacteria from different dietary sources for development of functional foods. Front. Microbiol..

[109] Lewis E.D., Antony J.M., Crowley D.C., Piano A., Bhardwaj R., Tompkins T.A., Evans M. (2020). Efficacy of *Lactobacillus paracasei* HA-196 and *Bifidobacterium longum* R0175 in Alleviating Symptoms of Irritable Bowel Syndrome (IBS): A Randomized, Placebo-Controlled Study. Nutrients.

[110] Cristofori F., Dargenio V.N., Dargenio C., Miniello V.L., Barone M., Francavilla R. (2021). Anti-Inflammatory and Immunomodulatory Effects of Probiotics in Gut Inflammation: A Door to the Body. Front. Immunol..

[111] Dobreva L., Atanasova N., Donchev P., Krumova E., Abrashev R., Karakirova Y., Mladenova R., Tolchkov V., Ralchev N., Dishliyska V. (2024). Candidate-Probiotic Lactobacilli and Their Postbiotics as Health-Benefit Promoters. Microorganisms.

[112] Diop L., Guillou S., Durand H. (2008). Probiotic food supplement reduces stress-induced gastrointestinal symptoms in volunteers: A double-blind, placebo-controlled, randomized trial. Nutr. Res..

[113] Gao H., Li X., Chen X., Hai D., Wei C., Zhang L., Li P. (2022). The Functional Roles of *Lactobacillus acidophilus* in Different Physiological and Pathological Processes. J. Microbiol. Biotechnol..

[114] Jang W.J., Lee J.M., Hasan T., Lee B.-J., Lim S.G., Kong I.-S. (2019). Effects of probiotic supplementation of a plant-based protein diet on intestinal microbial diversity, digestive enzyme activity, intestinal structure, and immunity in olive flounder (*Paralichthys olivaceus*). Fish Shellfish Immunol..

[115] Wei Z., He Z., Wang T., Wang X., Wang T., Long M. (2023). *Lactobacillus salivarius* WZ1 Inhibits the Inflammatory Injury of Mouse Jejunum Caused by Enterotoxigenic *Escherichia coli* K88 by Regulating the TLR4/NF-κB/MyD88 Inflammatory Pathway and Gut Microbiota. Microorganisms.

[116] Sun Y., Chen S., Ren F., Li Y. (2023). *Lactobacillus paracasei* N1115 attenuates obesity in high-fat diet-induced obese mice. Food Sci. Nutr..

[117] Ritze Y., Bárdos G., Claus A., Ehrmann V., Bergheim I., Schwiertz A., Bischoff S.C. (2014). Lactobacillus rhamnosus GG Protects against Non-Alcoholic Fatty Liver Disease in Mice. PLoS ONE.

[118] Ahn S.B., Jun D.W., Kang B.-K., Lim J.H., Lim S., Chung M.-J. (2019). Randomized, Double-blind, Placebo-controlled Study of a Multispecies Probiotic Mixture in Nonalcoholic Fatty Liver Disease. Sci. Rep..

[119] Wong V.W.-S., Vergniol J., Wong G.L.-H., Foucher J., Chan A.W.-H., Chermak F., Choi P.C.-L., Merrouche W., Chu S.H.-T., Pesque S. (2012). Liver Stiffness Measurement Using XL Probe in Patients With Nonalcoholic Fatty Liver Disease. Am. J. Gastroenterol..

[120] Park S.-S., Lim S.K., Lee J., Park H.K., Kwon M.-S., Yun M., Kim N., Oh Y.J., Choi H.-J. (2021). *Latilactobacillus sakei* WIKIM31 Decelerates Weight Gain in High-Fat Diet-Induced Obese Mice by Modulating Lipid Metabolism and Suppressing Inflammation. J. Microbiol. Biotechnol..

[121] Jang H.R., Park H.-J., Kang D., Chung H., Nam M.H., Lee Y., Park J.-H., Lee H.-Y. (2019). A protective mechanism of probiotic Lactobacillus against hepatic steatosis via reducing host intestinal fatty acid absorption. Exp. Mol. Med..

[122] Wang W., Shi L.P., Shi L., Xu L. (2018). Efficacy of probiotics on the treatment of non-alcoholic fatty liver disease. Zhonghua Nei Ke Za Zhi.

[123] Panpetch W., Phuengmaung P., Cheibchalard T., Somboonna N., Leelahavanichkul A., Tumwasorn S. (2021). Lacticaseibacillus casei Strain T21 Attenuates Clostridioides difficile Infection in a Murine Model Through Reduction of Inflammation and Gut Dysbiosis With Decreased Toxin Lethality and Enhanced Mucin Production. Front. Microbiol..

[124] Feng Y., Wang Y., Wang P., Huang Y., Wang F. (2018). Short-Chain Fatty Acids Manifest Stimulative and Protective Effects on Intestinal Barrier Function Through the Inhibition of NLRP3 Inflammasome and Autophagy. Cell. Physiol. Biochem..

[125] Karczewski J., Troost F.J., Konings I., Dekker J., Kleerebezem M., Brummer R.-J.M., Wells J.M. (2010). Regulation of human epithelial tight junction proteins by Lactobacillus plantarum in vivo and protective effects on the epithelial barrier. Am. J. Physiol. Liver Physiol..

[126] Johnson-Henry K.C., Donato K.A., Shen-Tu G., Gordanpour M., Sherman P.M. (2008). *Lactobacillus rhamnosus* Strain GG Prevents Enterohemorrhagic *Escherichia coli* O157:H7-Induced Changes in Epithelial Barrier Function. Infect. Immun..

[127] Chong P.L., Laight D., Aspinall R.J., Higginson A., Cummings M.H. (2021). A randomised placebo controlled trial of VSL#3® probiotic on biomarkers of cardiovascular risk and liver injury in non-alcoholic fatty liver disease. BMC Gastroenterol..

[128] Adams L.A., Wang Z., Liddle C., Melton P.E., Ariff A., Chandraratna H., Tan J., Ching H., Coulter S., De Boer B. (2020). Bile acids associate with specific gut microbiota, low-level alcohol consumption and liver fibrosis in patients with non-alcoholic fatty liver disease. Liver Int..

[129] Delik A., Dinçer S., Ülger Y., Akkız H., Karaoğullarından Ü. (2022). Metagenomic identification of gut microbiota distribution on the colonic mucosal biopsy samples in patients with non-alcoholic fatty liver disease. Gene.

[130] Hullar M.A.J., Jenkins I.C., Randolph T.W., Curtis K.R., Monroe K.R., Ernst T., Shepherd J.A., Stram D.O., Cheng I., Kristal B.S. (2021). Associations of the gut microbiome with hepatic adiposity in the Multiethnic Cohort Adiposity Phenotype Study. Gut Microbes.

[131] Xiang H., Sun D., Liu X., She Z.-G., Chen Y. (2022). The Role of the Intestinal Microbiota in Nonalcoholic Steatohepatitis. Front. Endocrinol..

[132] Jee J.J., Lim J., Park S., Koh H., Lee H.W. (2022). Gut microbial community differentially characterizes patients with nonalcoholic fatty liver disease. J. Gastroenterol. Hepatol..

[133] Yuan J., Chen C., Cui J., Lu J., Yan C., Wei X., Zhao X., Li N., Li S., Xue G. (2019). Fatty Liver Disease Caused by High-Alcohol-Producing Klebsiella pneumoniae. Cell Metab..

[134] Del Chierico F., Nobili V., Vernocchi P., Russo A., De Stefanis C., Gnani D., Furlanello C., Zandonà A., Paci P., Capuani G. (2017). Gut microbiota profiling of pediatric nonalcoholic fatty liver disease and obese patients unveiled by an integrated meta-omics-based approach. Hepatology.

[135] Leung H., Long X., Ni Y., Qian L., Nychas E., Siliceo S.L., Pohl D., Hanhineva K., Liu Y., Xu A. (2022). Risk assessment with gut microbiome and metabolite markers in NAFLD development. Sci. Transl. Med..

[136] Popov J., Despot T., Rodriguez D.A., Khan I., Mech E., Khan M., Bojadzija M., Pai N. (2024). Implications of Microbiota and Immune System in Development and Progression of Metabolic Dysfunction-Associated Steatotic Liver Disease. Nutrients.

[137] Nguyen H.T., Gu M., Werlinger P., Cho J.-H., Cheng J., Suh J.-W. (2022). *Lactobacillus sakei* MJM60958 as a Potential Probiotic Alleviated Non-Alcoholic Fatty Liver Disease in Mice Fed a High-Fat Diet by Modulating Lipid Metabolism, Inflammation, and Gut Microbiota. Int. J. Mol. Sci..

[138] Riezu-Boj J.I., Barajas M., Pérez-Sánchez T., Pajares M.J., Araña M., Milagro F.I., Urtasun R. (2022). *Lactiplantibacillus plantarum DSM20174* Attenuates the Progression of Non-Alcoholic Fatty Liver Disease by Modulating Gut Microbiota, Improving Metabolic Risk Factors, and Attenuating Adipose Inflammation. Nutrients.

[139] Wong V.W.S., Wong G.L.H., Chim A.M.L., Chu W.C.W., Yeung D.K.W., Li K.C.T., Chan H.L.Y. (2013). Treatment of nonalcoholic steatohepatitis with probiotics. A proof-of-concept study. Ann. Hepatol..

[140] Govindarajan K., MacSharry J., Casey P.G., Shanahan F., Joyce S.A., Gahan C.G.M. (2016). Unconjugated Bile Acids Influence Expression of Circadian Genes: A Potential Mechanism for Microbe-Host Crosstalk. PLoS ONE.

[141] Bourgin M., Kriaa A., Mkaouar H., Mariaule V., Jablaoui A., Maguin E., Rhimi M. (2021). Bile Salt Hydrolases: At the Crossroads of Microbiota and Human Health. Microorganisms.

[142] Kriaa A., Bourgin M., Potiron A., Mkaouar H., Jablaoui A., Gérard P., Maguin E., Rhimi M. (2019). Microbial impact on cholesterol and bile acid metabolism: Current status and future prospects. J. Lipid Res..

[143] Gu X.-C., Luo X.-G., Wang C.-X., Ma D.-Y., Wang Y., He Y.-Y., Li W., Zhou H., Zhang T.-C. (2014). Cloning and analysis of bile salt hydrolase genes from Lactobacillus plantarum CGMCC No. 8198. Biotechnol. Lett..

[144] Sridevi N., Vishwe P., Prabhune A. (2009). Hypocholesteremic effect of bile salt hydrolase from Lactobacillus buchneri ATCC 4005. Food Res. Int..

[145] Hu Y.-C., Ding X.-C., Liu H.-J., Ma W.-L., Feng X.-Y., Ma L.-N. (2024). Effects of *Lactobacillus paracasei* N1115 on gut microbial imbalance and liver function in patients with hepatitis B-related cirrhosis. World J. Gastroenterol..

[146] Qin N., Yang F., Li A., Prifti E., Chen Y., Shao L., Guo J., Le Chatelier E., Yao J., Wu L. (2014). Alterations of the human gut microbiome in liver cirrhosis. Nature.

[147] Ooi L.-G., Ahmad R., Yuen K.-H., Liong M.-T. (2010). Lactobacillus acidophilus CHO-220 and inulin reduced plasma total cholesterol and low-density lipoprotein cholesterol via alteration of lipid transporters. J. Dairy Sci..

[148] Jones M.L., Martoni C.J., Prakash S. (2012). Cholesterol lowering and inhibition of sterol absorption by Lactobacillus reuteri NCIMB 30242: A randomized controlled trial. Eur. J. Clin. Nutr..

[149] Jones M.L., Martoni C.J., Parent M., Prakash S. (2012). Cholesterol-lowering efficacy of a microencapsulated bile salt hydrolase-active *Lactobacillus reuteri* NCIMB 30242 yoghurt formulation in hypercholesterolaemic adults. Br. J. Nutr..

[150] Malpeli A., Taranto M.P., Cravero R.C., Tavella M., Fasano V., Vicentin D., Ferrari G., Magrini G., Hébert E., de Valdez G.F. (2015). Effect of Daily Consumption of Lactobacillus reuteri CRL 1098 on Cholesterol Reduction in Hypercholesterolemic Subjects. Food Nutr. Sci..

[151] Liong M., Shah N. (2005). Bile salt deconjugation ability, bile salt hydrolase activity and cholesterol co-precipitation ability of lactobacilli strains. Int. Dairy J..

[152] Chen Q., Wang S., Guo J., Xie Q., Evivie S.E., Song Y., Li B., Huo G. (2021). The Protective Effects of Lactobacillus plantarum KLDS 1.0344 on LPS-Induced Mastitis In Vitro and In Vivo. Front. Immunol..

[153] Kim B., Park K.-Y., Ji Y., Park S., Holzapfel W., Hyun C.-K. (2016). Protective effects of Lactobacillus rhamnosus GG against dyslipidemia in high-fat diet-induced obese mice. Biochem. Biophys. Res. Commun..

[154] Hu J., Hou Q., Zheng W., Yang T., Yan X. (2023). Lactobacillus gasseri LA39 promotes hepatic primary bile acid biosynthesis and intestinal secondary bile acid biotransformation. J. Zhejiang Univ. B.

[155] Li F., Jiang C., Krausz K.W., Li Y., Albert I., Hao H., Fabre K.M., Mitchell J.B., Patterson A.D., Gonzalez F.J. (2013). Microbiome remodelling leads to inhibition of intestinal farnesoid X receptor signalling and decreased obesity. Nat. Commun..

[156] Degirolamo C., Rainaldi S., Bovenga F., Murzilli S., Moschetta A. (2014). Microbiota Modification with Probiotics Induces Hepatic Bile Acid Synthesis via Downregulation of the Fxr-Fgf15 Axis in Mice. Cell Rep..

[157] Nabavi S., Rafraf M., Somi M., Homayouni-Rad A., Asghari-Jafarabadi M. (2014). Effects of probiotic yogurt consumption on metabolic factors in individuals with nonalcoholic fatty liver disease. J. Dairy Sci..

[158] Tsui K., Yen T., Huang C., Hong K. (2021). *Lactobacillus rhamnosus* GG as dietary supplement improved survival from lipopolysaccharides-induced sepsis in mice. Food Sci. Nutr..

[159] Tomaro-Duchesneau C., Jones M.L., Shah D., Jain P., Saha S., Prakash S. (2014). Cholesterol Assimilation by *Lactobacillus* Probiotic Bacteria: An In Vitro Investigation. BioMed Res. Int..

[160] Tsai C.-C., Lin P.-P., Hsieh Y.-M., Zhang Z.-Y., Wu H.-C., Huang C.-C. (2014). Cholesterol-Lowering Potentials of Lactic Acid Bacteria Based on Bile-Salt Hydrolase Activity and Effect of Potent Strains on Cholesterol Metabolism In Vitro and In Vivo. Sci. World J..

[161] Kim D., Jeong D., Kang I., Kim H., Song K., Seo K. (2017). Dual function of *Lactobacillus kefiri* DH5 in preventing high-fat-diet-induced obesity: Direct reduction of cholesterol and upregulation of PPAR-α in adipose tissue. Mol. Nutr. Food Res..

[162] Nakamura F., Ishida Y., Sawada D., Ashida N., Sugawara T., Sakai M., Goto T., Kawada T., Fujiwara S. (2016). Fragmented Lactic Acid Bacterial Cells Activate Peroxisome Proliferator-Activated Receptors and Ameliorate Dyslipidemia in Obese Mice. J. Agric. Food Chem..

[163] Zhao Z., Wang C., Zhang L., Zhao Y., Duan C., Zhang X., Gao L., Li S. (2019). Lactobacillus plantarum NA136 improves the non-alcoholic fatty liver disease by modulating the AMPK/Nrf2 pathway. Appl. Microbiol. Biotechnol..

[164] Hardie D.G., Pan D.A. (2002). Regulation of fatty acid synthesis and oxidation by the AMP-activated protein kinase. Biochem. Soc. Trans..

[165] Saponaro C., Gaggini M., Carli F., Gastaldelli A. (2015). The Subtle Balance between Lipolysis and Lipogenesis: A Critical Point in Metabolic Homeostasis. Nutrients.

[166] Smith B.K., Marcinko K., Desjardins E.M., Lally J.S., Ford R.J., Steinberg G.R. (2016). Treatment of nonalcoholic fatty liver disease: Role of AMPK. Am. J. Physiol. Endocrinol. Metab..

[167] Quan H.Y., Kim D.Y., Kim S.J., Jo H.K., Kim G.W., Chung S.H. (2013). Betulinic acid alleviates non-alcoholic fatty liver by inhibiting SREBP1 activity via the AMPK–mTOR–SREBP signaling pathway. Biochem. Pharmacol..

[168] Ivey K.L., Hodgson J.M., Kerr D.A., Thompson P.L., Stojceski B., Prince R.L. (2015). The effect of yoghurt and its probiotics on blood pressure and serum lipid profile; a randomised controlled trial. Nutr. Metab. Cardiovasc. Dis..

[169] Famouri F., Shariat Z., Hashemipour M., Keikha M., Kelishadi R. (2017). Effects of Probiotics on Nonalcoholic Fatty Liver Disease in Obese Children and Adolescents. J. Pediatr. Gastroenterol. Nutr..

[170] Aller R., De Luis D.A., Izaola O., Conde R., Gonzalez Sagrado M., Primo D., Fuent B.D.L., Gonzalez J. (2011). Effect of a probiotic on liver aminotransferases in nonalcoholic fatty liver disease patients: A double blind ran-domized clinical trial. Eur. Rev. Med. Pharmacol. Sci..

[171] Kobyliak N., Abenavoli L., Mykhalchyshyn G., Kononenko L., Boccuto L., Kyriienko D., Dynnyk O. (2018). A Multi-strain Probiotic Reduces the Fatty Liver Index, Cytokines and Aminotransferase levels in NAFLD Patients: Evidence from a Randomized Clinical Trial. J. Gastrointest. Liver Dis..

[172] Ejtahed H., Mohtadi-Nia J., Homayouni-Rad A., Niafar M., Asghari-Jafarabadi M., Mofid V., Akbarian-Moghari A. (2011). Effect of probiotic yogurt containing Lactobacillus acidophilus and Bifidobacterium lactis on lipid profile in individuals with type 2 diabetes mellitus. J. Dairy Sci..

[173] Toshimitsu T., Gotou A., Sashihara T., Furuichi K., Hachimura S., Shioya N., Suzuki S., Asami Y. (2021). Ingesting Yogurt Containing Lactobacillus plantarum OLL2712 Reduces Abdominal Fat Accumulation and Chronic Inflammation in Overweight Adults in a Randomized Placebo-Controlled Trial. Curr. Dev. Nutr..

[174] Kumar V. (2020). Toll-like receptors in sepsis-associated cytokine storm and their endogenous negative regulators as future immunomodulatory targets. Int. Immunopharmacol..

[175] Bakhshimoghaddam F., Shateri K., Sina M., Hashemian M., Alizadeh M. (2018). Daily Consumption of Synbiotic Yogurt Decreases Liver Steatosis in Patients with Nonalcoholic Fatty Liver Disease: A Randomized Controlled Clinical Trial. J. Nutr..

[176] Loguercio C., Federico A., Tuccillo C., Terracciano F., D’Auria M.V., De Simone C., Del Vecchio Blanco C. (2005). Beneficial Effects of a Probiotic VSL#3 on Parameters of Liver Dysfunction in Chronic Liver Diseases. J. Clin. Gastroenterol..

[177] Rajkumar H., Mahmood N., Kumar M., Varikuti S.R., Challa H.R., Myakala S.P. (2014). Effect of Probiotic (VSL#3) and Omega-3 on Lipid Profile, Insulin Sensitivity, Inflammatory Markers, and Gut Colonization in Overweight Adults: A Randomized, Controlled Trial. Mediat. Inflamm..

[178] Noh S.Y., Kang S.-S., Yun C.-H., Han S.H. (2015). Lipoteichoic acid from Lactobacillus plantarum inhibits Pam2CSK4-induced IL-8 production in human intestinal epithelial cells. Mol. Immunol..

[179] Duseja A., Acharya S.K., Mehta M., Chhabra S., Shalimar, Rana S., Das A., Dattagupta S., Dhiman R.K., Chawla Y.K. (2019). High potency multistrain probiotic improves liver histology in non-alcoholic fatty liver disease (NAFLD): A randomised, double-blind, proof of concept study. BMJ Open Gastroenterol..

[180] Santos A.A., Duarte R., Duarte M., Arella F., Marques V., Roos S., Rodrigues C.M. (2024). Impact of Lactobacillaceae supplementation on the multi-organ axis during MASLD. Life Sci..

[181] Saenz E., Montagut N.E., Wang B., Stein-Thöringer C., Wang K., Weng H., Ebert M., Schneider K.M., Li L., Teufel A. (2024). Manipulating the Gut Microbiome to Alleviate Steatotic Liver Disease: Current Progress and Challenges. Engineering.

[182] Mijangos-Trejo A., Nuño-Lambarri N., Barbero-Becerra V., Uribe-Esquivel M., Vidal-Cevallos P., Chávez-Tapia N. (2023). Prebiotics and Probiotics: Therapeutic Tools for Nonalcoholic Fatty Liver Disease. Int. J. Mol. Sci..

[183] Rastogi S., Singh A. (2022). Gut microbiome and human health: Exploring how the probiotic genus Lactobacillus modulate immune responses. Front. Pharmacol..

[184] Ghosh S.S., Wang J., Yannie P.J. (2020). Intestinal Barrier Dysfunction, LPS Translocation, and Disease Development. J. Endocr. Soc..

[185] Didari T., Solki S., Mozaffari S., Nikfar S., Abdollahi M. (2014). A systematic review of the safety of probiotics. Expert Opin. Drug Saf..

[186] Zhou X., Wang J., Zhou S., Liao J., Ye Z., Mao L. (2023). Efficacy of probiotics on nonalcoholic fatty liver disease: A meta-analysis. Medicine.

[187] Rannikko J., Holmberg V., Karppelin M., Arvola P., Huttunen R., Mattila E., Kerttula N., Puhto T., Tamm Ü., Koivula I. (2021). Fungemia and Other Fungal Infections Associated with Use of *Saccharomyces boulardii* Probiotic Supplements. Emerg. Infect. Dis..

[188] Cerk K., Aguilera-Gómez M. (2022). Microbiota analysis for risk assessment: Evaluation of hazardous dietary substances and its potential role on the gut microbiome variability and dysbiosis. EFSA J..

[189] Singh R.P., Shadan A., Ma Y. (2022). Biotechnological Applications of Probiotics: A Multifarious Weapon to Disease and Metabolic Abnormality. Probiotics Antimicrob. Proteins.

